# Implication of the micro- and lithofacies types on the quality of a gas-bearing deltaic reservoir in the Nile Delta, Egypt

**DOI:** 10.1038/s41598-023-35660-0

**Published:** 2023-06-01

**Authors:** Bassem S. Nabawy, Emad A. Abd El Aziz, Mohamed Ramadan, Amer A. Shehata

**Affiliations:** 1 Geophysical Sciences Department, National Research Centre, Cairo, Egypt; 2 Geological and Geophysical Engineering Department, Faculty of Petroleum and Mining Engineering, Suez University, Suez, Egypt; 3 Geology Department, Faculty of Science, Port Said University, Port Said, 42522 Egypt; 4 Department of Physical and Environmental Sciences, College of Science, Texas A&M University-Corpus Christi, 6300 Ocean Drive, Corpus Christi, TX 78412 USA

**Keywords:** Core processes, Geology

## Abstract

The Messinian Qawasim sequence, as one of the most important reservoirs in the Egyptian Nile Delta, represents a typical gas-bearing deltaic reservoir sequence. It aims at delineating the implication of the litho- and microfacies associations on the reservoir characteristics of the different stages of the fluvial deltas including the prodelta, proximal/distal delta front, and the delta plain depositional sequences. The studied reservoir sequence was divided into two units; upper and lower clastic units. The petrophysical properties of these two units were studied on the borehole scale using the gamma-ray, caliper, sonic, density, neutron, and resistivity logs to estimate the reservoir parameters including the total and effective porosities, water saturation, shale volume, and the net-pay thickness. For more details, they are also examined on the plug-scale using the core dataset including helium porosity, density, permeability, and fluids saturations, where the flow zone indicator, the reservoir potentiality index, the reservoir quality index, and the average reservoir pore radius were then estimated. The entire sequence is divided into five reservoir rock types (RRT1-RRT5) where, the best quality is assigned to the first RRT (the upper reservoir unit), and the lowest quality is assigned to the RRT5 of the lower reservoir unit. Based on core description and the petrographical studies five lithofacies and four microfacies have been identified. The lithofacies are (1) laminated mudstones/siltstones, (2) ripple laminated sandstones, (3) cross-laminated sandstones, (4) cross-bedded sandstones, and (5) pebbly massive sandstones. These lithofacies are primarily composed of four microfacies; sublithic arenites, subarkose arenite, glauconitic quartz wacke, and sandy mudstone/siltstone. Based on this study, the pebbly massive and the cross-bedded sandstones of the upper unit which is composed of the sublithic and subarkose arenites (RRT1-RRT2) have the best reservoir quality. On the other hand, the lowest quality is assigned to the RRT5 (sandy mudstone/siltstone microfacies) which is represented on the macro scale by the laminated mudstones/siltstones lithofacies. The integration between multi-scale datasets (core-well, petrography, well logs, and seismic) gives a precise picture of the deltaic Qawasim reservoir rock units in the Nile Delta. This workflow has never been applied to the deltaic system in the onshore Nile delta and North Africa. Thereby, this study is considered a standard case study for the deltaic sequences and its proposed workflow is applicable to the Nile Delta elsewhere and worldwide for similar reservoir sequences.

## Introduction

During the last decades, the Nile Delta became one of the most important prolific gas producing provinces in Egypt. The majority of gas production of the Nile delta province is mainly attributed by the Miocene and Pliocene reservoirs which have approximate reserves reaching 60 TCF^1^. The Messinian sequences (Abu Madi and Qawasim Formations) contain the major hydrocarbon producing reservoirs^1,2^. The Qawasim Formation (the target of this study) was interpreted as Eonile deltaic deposits and has been developed prior to the Messinian evaporites^3,4^. The Qawasim Formation has been distinguished as oil and gas-bearing reservoirs in the West Dikirnis Field^4–6^. Interpreting the depositional environmental setting, the reservoir lithofacies, and various petrophysical datasets figure out a precise picture of the reservoir’ quality and reduces the risk and the uncertainty level of hydrocarbon exploration and the future development plans^7–14^. These integrations clearly illustrate the importance of constructing an accurate depositional model for understanding of the reservoir properties distribution in this field. The lateral variation, spatial orientation, net-gross, reservoir volume, fluids saturation, and the other petrophysical characters of the Qawasim reservoir are all governed by the different processes of transportation and deposition/climatic variations, structural framework/tectonic evolution, basin configuration, and the sea-level fluctuations.

The importance of this study is due to the scarcity of studies on the impact of both the depositional facies and the petrophysical reservoir characteristics on the hydrocarbon potentiality of the Messinian reservoir in the West Dikirnis Oil Field. Therefore, the aim of the present study is focused on the differential impact of the lithofacies, microfacies, and the petrophysical characters (permeability, porosity and fluid saturations) on the hydrocarbons potentiality, flow capacity, and the storage capacity of the Messinian reservoirs.

## Geologic setting

### Structural setting

#### Regional structural setting of Nile Delta

**Figure 1:**
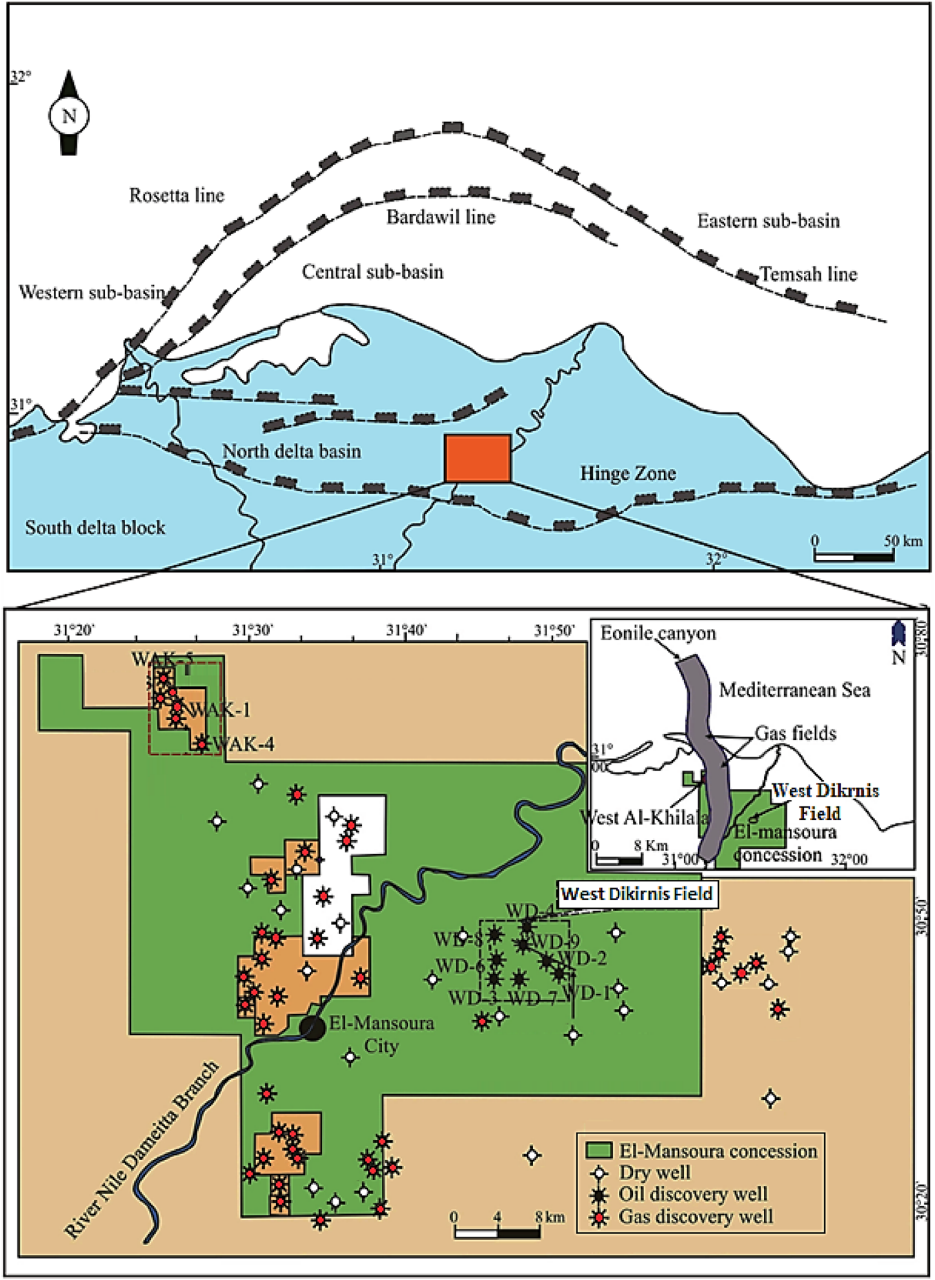
Location map showing West Dikirnis Oil Field, Nile Delta province, Egypt [4].

The Nile Delta is situated in the extreme northern region of Egypt along 1000 km of the Mediterranean coast line (Fig. 1). It has been affected by three main tectonic events: (1) the folding of Syrian Arc system, (2) the Rifting of Red Sea, and (3) the African-Anatolian plate^3,17^. The sedimentary facies distribution along the Nile Delta is structurally-controlled by a Hinge Zone that formed the most significant structural feature in this area^3,15,16^. The Hinge-Zone is crossing through the middle delta region and represents a faulted E-W flexure zone. It is related to the rifting phase of the African/Arabian plates and the separation from the Eurasian plate during the Jurassic time where the crustal-breakup of the southern Neotethys is happened^3^. This zone is structurally separates the Nile Delta into two major sedimentary provinces; southern and northern provinces (Fig. 1). The Hinge Zone is considered a facies and structural boundary separated the Northern Delta Tertiary basinal facies from the Southern Delta Cretaceous-Eocene platform^18^. Furthermore, along that zone a continuous subsidence resulting in a rapid increase in thickness of the Tertiary sediments inflow in the Northern Delta Basin^19^. Tectonically, the Southern Delta Basin is situated on the Egyptian unstable shelf while the Northern Deltaic block is steeply intersected and located in the continental stable shelf^20^. During the Neogene and the Late Cretaceous age, the Rosetta and Temsah fault trends dissected the Northern Nile Delta Basin and divided it into three sub-basins: central, eastern and western ones (Fig. 1)^18,20^.

#### Structural setting of West Dikirnis oil field

**Figure 2:**
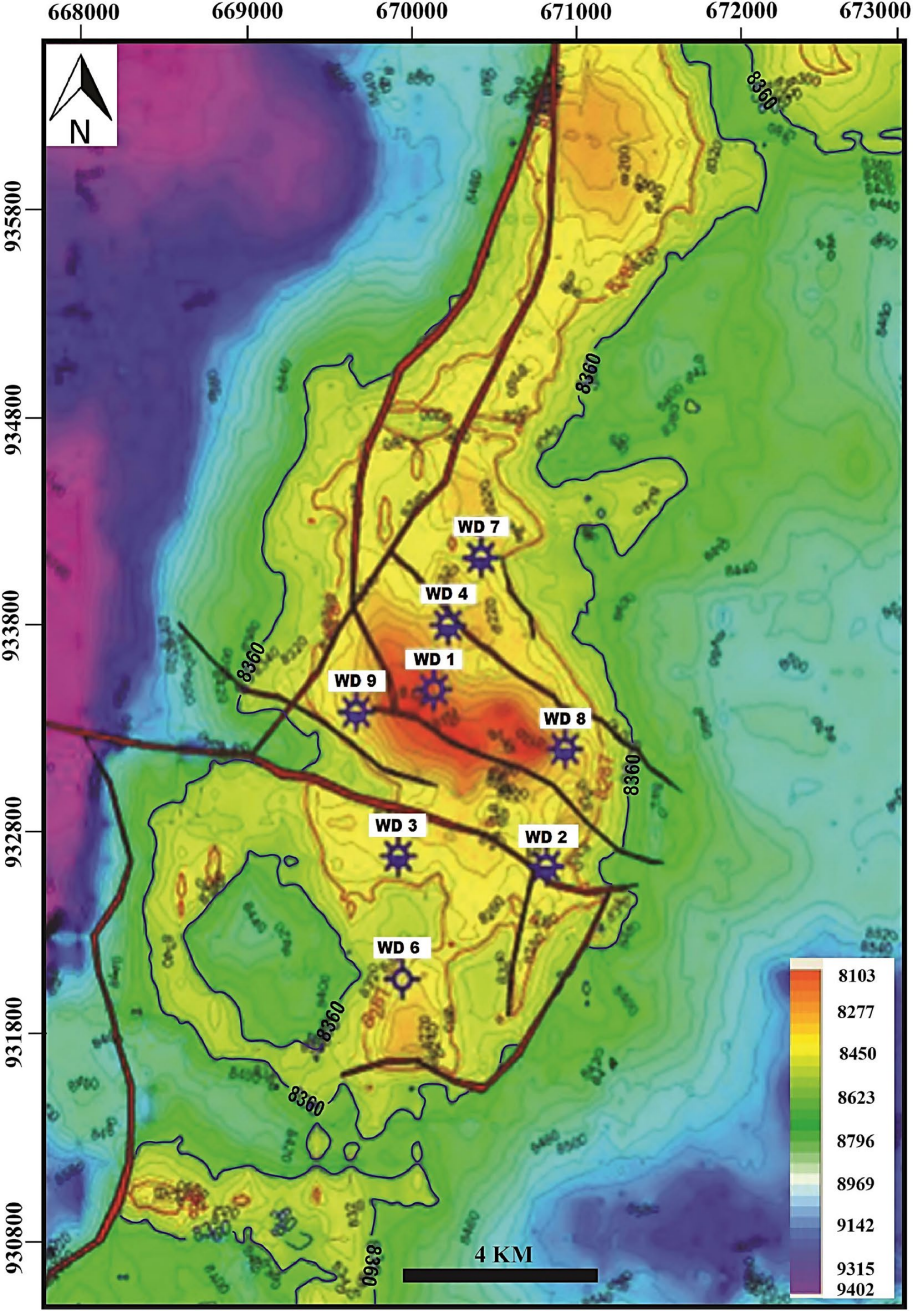
Structure contour map on top Qawasim reservoir, W. Dikirnis Field, onshore Nile Delta, Egypt (depths in feet, C.I. = 86.57 ft). The map was created using Petrel software (v 2017).

**Figure 3:**
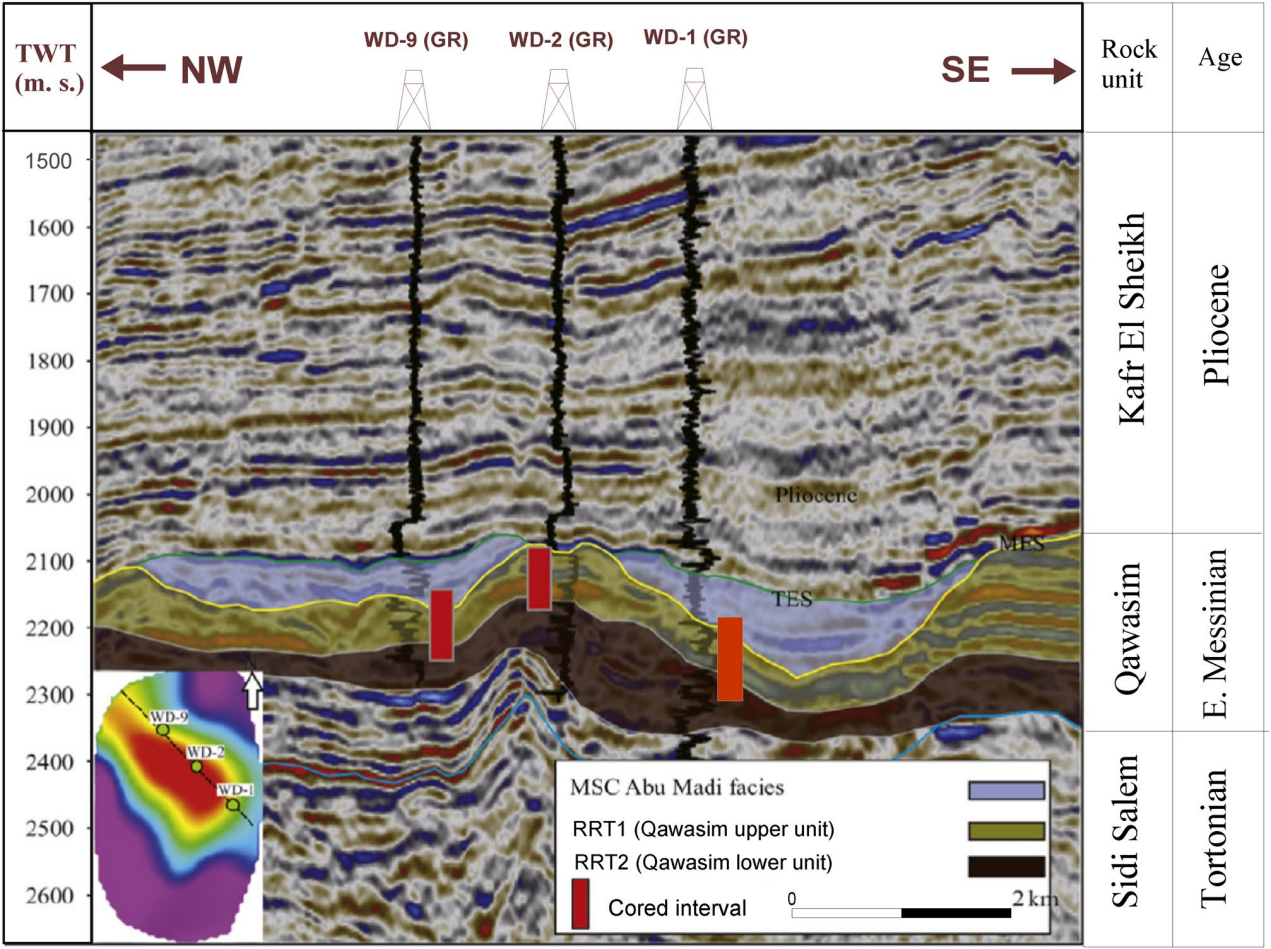
NW–SE seismic profile along West Dikirnis Field illustrating the different seismic stratigraphic zones within the pre-Messinian salinity crisis Qawasim Formation [4].

The studied West Dikirnis Field is situated in the onshore of the eastern part of El Mansoura Concession, in the Nile Delta central sub-basin, Egypt, in between latitudes:30° 05′ & 30° 08′ N and longitudes: 31° 34′ & 31° 36′ E and cover about 17.5 km^2^ (Fig. 1). This sub-basin includes thick Tertiary sediments sequence which represents the most prospective reservoirs and hydrocarbon source rocks^21^. The structure contour map of the top Qawasim reservoir surface (Fig. 2) revealed that the study area is mainly dissected by a hinge line that takes the E–W major fault trends throwing down the Tertiary sequence to the north with some minor faults trending through the N–S Baltim trend throwing down the sequence to the east and west^17^. These faults patterns caused a creation of a four-way dip closure of the structural high of the West Dikirnis Field that trending WNW-ESE, and had been also dissected by many sets of minor faults trending E–W and NE–SW^15^. These structural highs are the most prominent feature in the West Dikirnis Field (Figs. 2, 3), where the active syn-depositional tectonism have had a controlling role on the distribution of the promising Messinian sandstone reservoirs. Also, the presented NW–SE seismic profile (Fig. 3) helped in displaying the fault pattern. This allowed improving our understanding of the structural framework of West Dikirnis Field combined with the results of the borehole data processing and analysis. The deeply-seated faults (trending parallel to the hinge-zone) are suitable for the hydrocarbon entrapment while the underlying Early Tertiary, Cretaceous and Jurassic sediments are considered a good source rock for hydrocarbons generation^4^.

### Lithostratigraphic setting

**Figure 4:**
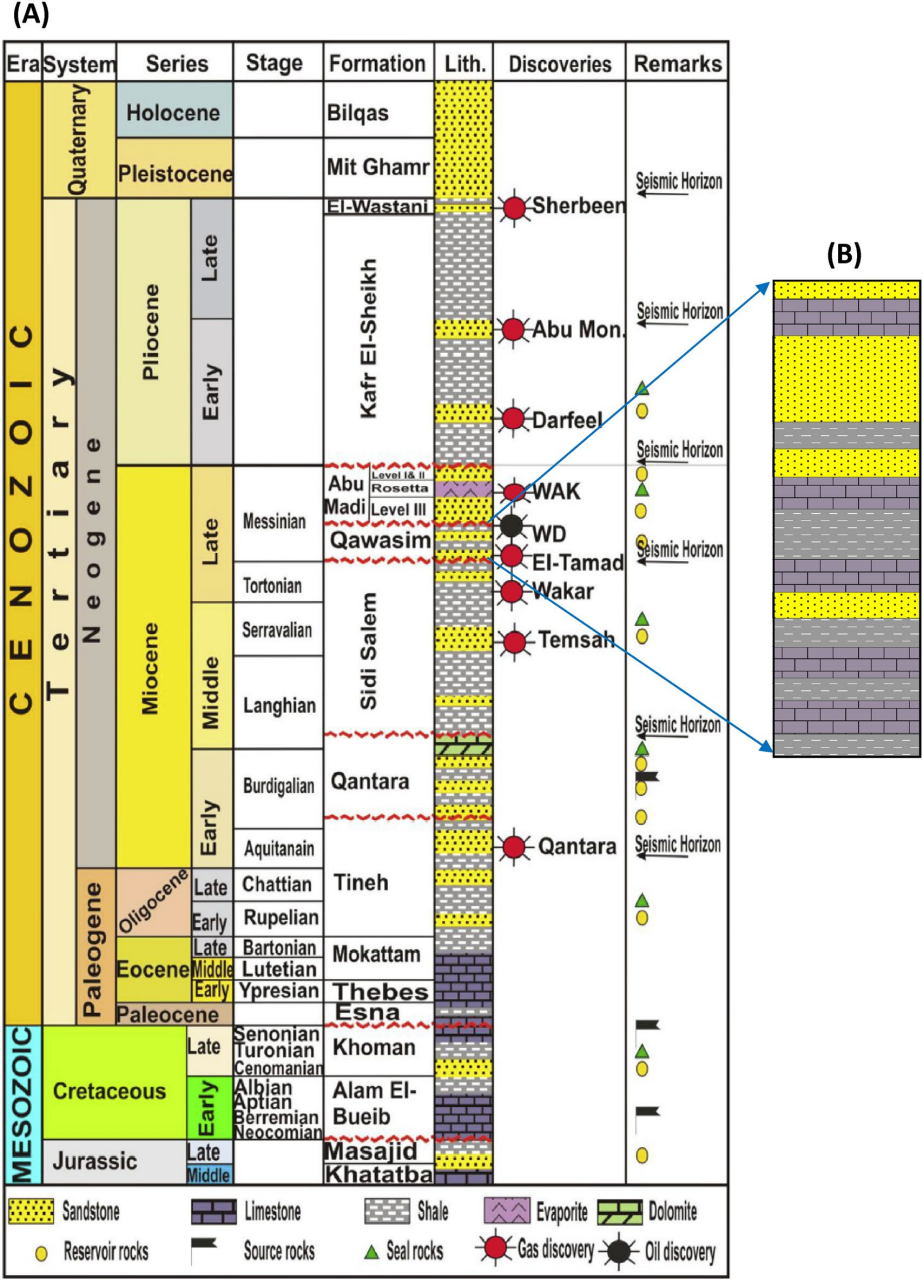
(**A**) Generalized stratigraphic column of onshore Nile Delta province in Egypt, and (**B**) Detailed stratigraphic section of the Qawasim Formation.

By the beginning of the Oligocene, the alternations of coarse clastic fluvial facies and the open marine muddy facies dominated in the Tineh Formation^3,17,22–25^. Upwards, the Miocene-Pliocene succession (the Miocene Qantara, Sidi Salem, Qawasim, and Abu Madi Formations, as well as the Pliocene Kafr El-Sheikh, and El-Wastani Formations, Fig. 4A) is characterized by the presence of two major regional unconformities which were caused by crucial changes in the sedimentation rates and paleobathymetry of the Delta basin^17^. The Middle and Late Miocene strata are separated by the first unconformity in between the Qantara and Sidi Salem Formations, whereas the Late Miocene sediments ended by another unconformity which refers to the major Messinian salinity crisis event in the Nile Delta in between the Messinian Abu Madi and the Pliocene Kafr El-Sheikh Formations (Fig. 4). The Nile Delta basin is invaded by a Marine transgression that has been dominated during the Early Pliocene Kafr El-Sheikh Formation (Zanclean time); this Marine transgression continued till the Late Pliocene El-Wastani Formation which was discriminated by a climatic changes period and accompanied by permanently depositional system changing from fluvio-marine to fluviatile-dominated^3,16^. In general, the Neogene lithostratigraphic sequence of the West Dikirnis Field encompasses, from top to bottom, the Pleistocene to Recent Mit Ghamr Formation, the Pleistocene El Wastani Formation, the Pliocene Kafr El Sheikh Formation, the Messinian Qawasim Formation, and the Tortonian Sidi Salim Formation (Fig. 4).

## Materials and methods

The study of Qawasim Formation in West Dikirnis Field is based on well logging, petrographic and core data sets of four wells (WD-1, WD-2, WD-3, and WD-7) in the West Dikirnis Field (Fig. 1). The available well logging datasets encompass an entire set of borehole logging data (gamma-ray, resistivity, density, neutron, PE, and sonic logs). Logging data are available to depth intervals for 8170–8320 feet for WD-1, 8270–8450 ft for WD-2, 8350–8410 ft for WD-3, and 9040–9170 ft for WD-7 wells.

The borehole logging data was interpreted using the Interactive Petrophysics (IP) software (v 3.6) to evaluate the petrophysical and reservoir properties of the examined clastic sequence of the Qawasim Formation. The petrophysical evaluation of the studied Qawasim reservoir has been carried out by analyzing and presenting the different petrophysical values that derived from the well log data processing which involves porosity, water saturation, permeability and shale volume estimation. For the present study, the total porosity (∅_T_) was estimated from logs using a combined neutron-density method^26^, while the shale volume (Vsh) was calculated based on the gamma-ray log.

1 $$ \emptyset_{{\text{T}}} = \surd (\emptyset_{{\text{D}}}^{2} + \emptyset_{{\text{N}}}^{2} ) $$

where ∅_D_ is the density porosity and ∅_N_ is the neutron porosity.

Then, the effective porosity (∅e) was estimated from logs by correcting the total porosity (∅_T_) for the shale volume as follows.

2 $$ \emptyset {\text{e}} = \emptyset_{{\text{T}}} \times \left( {{1} - {\text{Vsh}}} \right) $$

Then, the effective porosity was estimated by eliminating the shale volume impact from total porosity. Finally, the water saturation was calculated using Archie's equations^27^.

Consequently, the net-pay thickness of the studied reservoir in the different wells were estimated considering the accumulative thickness of the entire promising zones in each well and considering the bulk density of shale equals 2.54 g/cc, 21.5% for its neutron porosity, 79.5 μsec/ft for its transit time, and 3.1 Ω.m for its true electric resistivity (Table 1). On the other side, the applied Archie’s parameters including a, m, n and Rw are equal to 1.0, 2.1, 2.3, and 0.021 Ω.m, respectively (at 215 °F formation temperature). These parameters were estimated using the Pickett plot. Eventually, the promising zones are those that have porosity higher than 5% (∅e) as cutoff value, and contain shale volume and water saturation lower than 40% (Vsh) and 65% (Sw), respectively. These values were estimated based on integration between the production and the petrophysical data.

**Table 1:** The reservoir parameters derived from the well log data of the Qawasim Formation, West Dikirnis Field.

Well	Units	Top (ft)	Bottom (ft)	Gross (ft)	Net (ft)	N/G	∅_eff_	Sw	Vsh	Phi*H	Phi*Sw	Phi*So*H
WD-1	Unit-I	8171	8232	61	59.75	0.98	0.31	0.081	0.05	18.52	0.03	17.03
	Unit-II	8232	8315	83	36.5	0.44	0.222	0.297	0.099	8.12	0.07	5.71
WD-2	Unit-I	8266	8397	131	130.3	0.994	0.22	0.162	0.02	28.7	0.04	24.06
	Unit-II	8397	8451	54	1.0	0.019	0.145	0.567	0.381	0.15	0.08	0.06
WD-3	Unit-I	8355	8397	42	Dry	–	–	–	–	–	–	–
	Unit-II	8397	8410	13	Dry	–	–	–	–	–	–	–
WD-7	Unit-I	9046	9116	70	58.5	0.836	0.32	0.329	0.098	13.56	0.08	9.09
	Unit-II	9116	9160	44	6.0	0.136	0.252	0.524	0.11	1.51	0.13	0.72

where N/G is the net to gross ratio, Phi or ∅_eff_ is the effective porosity, Sw is the water saturation ratios, Vsh is the shale volume, and H is the thickness of the net pay zone. The blank cells refer to dry well, so no reservoir was assigned and not net pay thickness was estimated. Therefore, the other reservoir parameters were not estimated.

Besides, parallel to the well log analysis, the structure contour maps were created using Petrel software (v 2017). The core data of the Qawasim Formation is represented by a total of 435 plug samples (170 samples from WD-2 well, 164 samples from WD-3 well, and 101 samples from WD-7 well) selected systematically one plug/foot and representatively for each lithofacies. The cored intervals vary between 8267 and 9150 feet for the various wells. The plugged samples were then cleaned using soxhlet apparatus filled with mixture of solvents (Toluene and methanol). The cleaning efficiency was checked using UV light and silver nitrates.

The measured core data is represented by the grain density (ρ_g_), bulk density (ρ_b_), vertical and horizontal permeabilities (k_V_& k_H_, respectively), helium porosity (∅_He_), and fluids saturations (water, oil, and gas).

Measuring the bulk density (ρ_b_ in g/cc) was carried out using direct measurements for the bulk volume and the dry weight (Vb and wt), while the grain density (ρ_g_ in g/cc) and the helium porosity (∅e in decimals) were measured using a Quantachrome helium pycnometer at 14.5 psi which estimates the grain volume (Vg).

3 $$\uprho _{{\text{b}}} = {\text{wt}}/{\text{Vb}},\uprho _{{\text{g}}} = {\text{wt}}/{\text{Vg}} $$

4 $$ (\emptyset {\text{e}}) = {1}00 \times \left( {{\text{Vb}} - {\text{Vg}}} \right)/{\text{Vb}}\quad[{28, 29]} $$

Permeability (_H_ in md) of the different samples was measured for two plugs drilled in two directions at each depth; in horizontal and vertical directions, to get the horizontal (k_H_ in md) and vertical permeability (k_V_ in md); i.e., there are two permeability values corresponding to each porosity reading. Measuring the permeability was applied using a nitrogen gas permeameter considering the flow rate (Q), viscosity (μ), L and A are the length and cross-sectional area of the measured plug, and ΔP is the applied pressure difference. After that, the anisotropy of permeability (λ_k_) has been calculated as follows.

5 $$ {\text{k}} = ({\text{Q}}.{\upmu}.{\text{L}})/({\text{A}}.\Delta{\text{P}})\quad {\text{Dracy}^{\prime {\rm s}}}\,{\text{law }}\quad\left[ {{3}0} \right] $$

6 $$  \lambda  _{{\text{k}}} =  {\text{  SQRT}}\left( {{\text{k}}_{{\text{H}}} /{\text{k}}_{{\text{V}}} } \right)\quad \left[ {{31} - {33}} \right] $$

The anisotropy of permeability is an important parameter indicates the ability of the fluids to flow horizontally and vertically inside the reservoir, i.e., its ability to produce hydrocarbons. It is also, a critical parameter which indicates the possibility of water and gas coning around the borehole during the hydrocarbons production.

The fluids saturation values were measured using the mercury porosimeter at 50 psi for measuring the bulk volume of the studied samples and at 700 psi for measuring the gas volume. Besides, the retort oven was used to estimate the water volume at 120 °C and the oil volume at 650 °C. Then the fluids saturation was estimated considering the bulk, gas, water, and oil volumes.

For this study, the hydraulic flow unit (HFU) concept was firstly introduced by Amaefule et al.^34^ and applied by many authors^35,36^. It is applied for evaluating the Qawasim Formation’ reservoir quality in the studied Field. This concept is based on the flow zone indicator (FZI), normalized porosity index (NPI), and reservoir quality index (RQI) parameters which can be calculated using the following mathematical models.

7 $$ {\text{FZI}} = {\text{RQI}}/{\text{NPI}}\quad {[34]} $$

8 $$ {\text{NPI}} = \emptyset /({1} - \emptyset ) $$

9 $$ {\text{RQI}} = 0.0{314} \times {\text{SQRT}}({\text{k}}/\emptyset ) $$

where k is the permeability in md, and ∅ is porosity in decimals.

Following the reservoir quality classification ranks of (Table 3), the reservoir quality ranks of the different samples were estimated based on the FZI and the RQI values and the reservoir potentiality index (RPI) values were estimated using the following equation.

10 $$ {\text{RPI}} = \left( {{\text{RQI}}_{{{\text{rank}}}} + {\text{FZI}}_{{{\text{rank}}}} } \right)/{2}\quad {[37, 38]} $$

Plotting these parameters as X–Y plots enable dividing samples into reservoir rock types (RRTs), each rock type has their diagnostic petrophysical and reservoir parameters and its mineral composition as well. The petrophysical behavior of rock types is always controlled by their mineral composition, whether they are carbonates or clastics, fine or coarse-grained, well or badly-sorted, and whether they are cemented by silica, argillaceous or calcareous cement. Considering the FZI and RQI values, the reservoir quality parameters were classified into different ranks considering the classification ranks of Nabawy et al.^37,38^, where tight samples rocks have the 6th rank while the excellent reservoir rocks have the 1st rank^37–39^.

The core data measuring techniques and their workflow were explained in detail by many authors^10,11,37,39–46^.

For the petrographical examination, a number of thin-sections were prepared by impregnating the sliced rock chips with blue-dyed araldite for describing the different pore types. The impregnated thin sections were examined using a polarized microscope to identify their textural and mineralogical compositions. The classification and terminologies of the clastic samples were followed the sandstone classification of Pettijohn et al.^47^.

## Results

### Petrophysical well logging analysis

**Figure 5:**
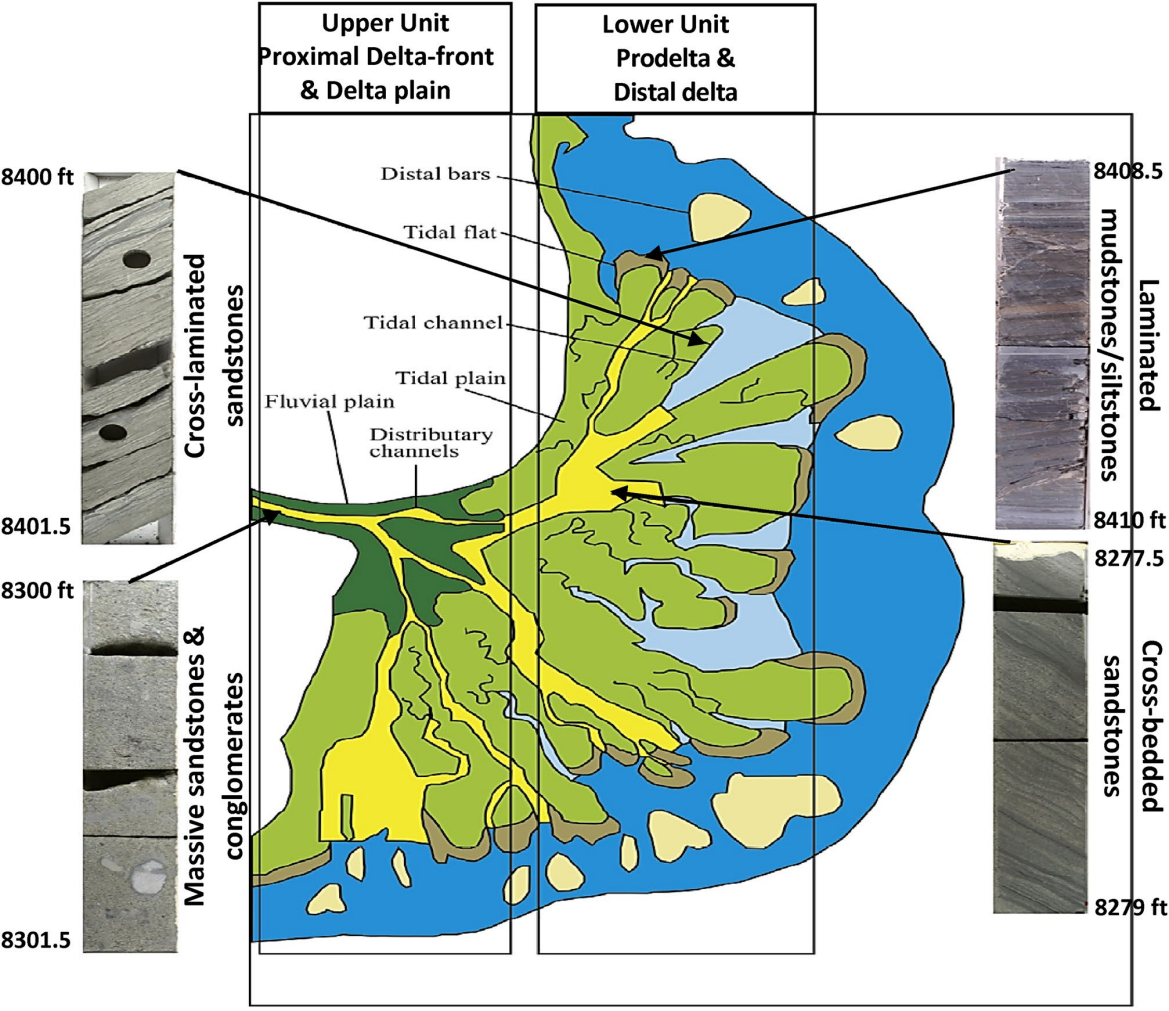
Conceptual model of the deltaic Qawasim Formation and core photos for the corresponding sedimentary facies, WD-2 well, West Dikirnis Field, onshore Nile Delta (modified after Leila and Moscariello [4], (Core scale = 1.5 feet/image).

**Figure 6:**
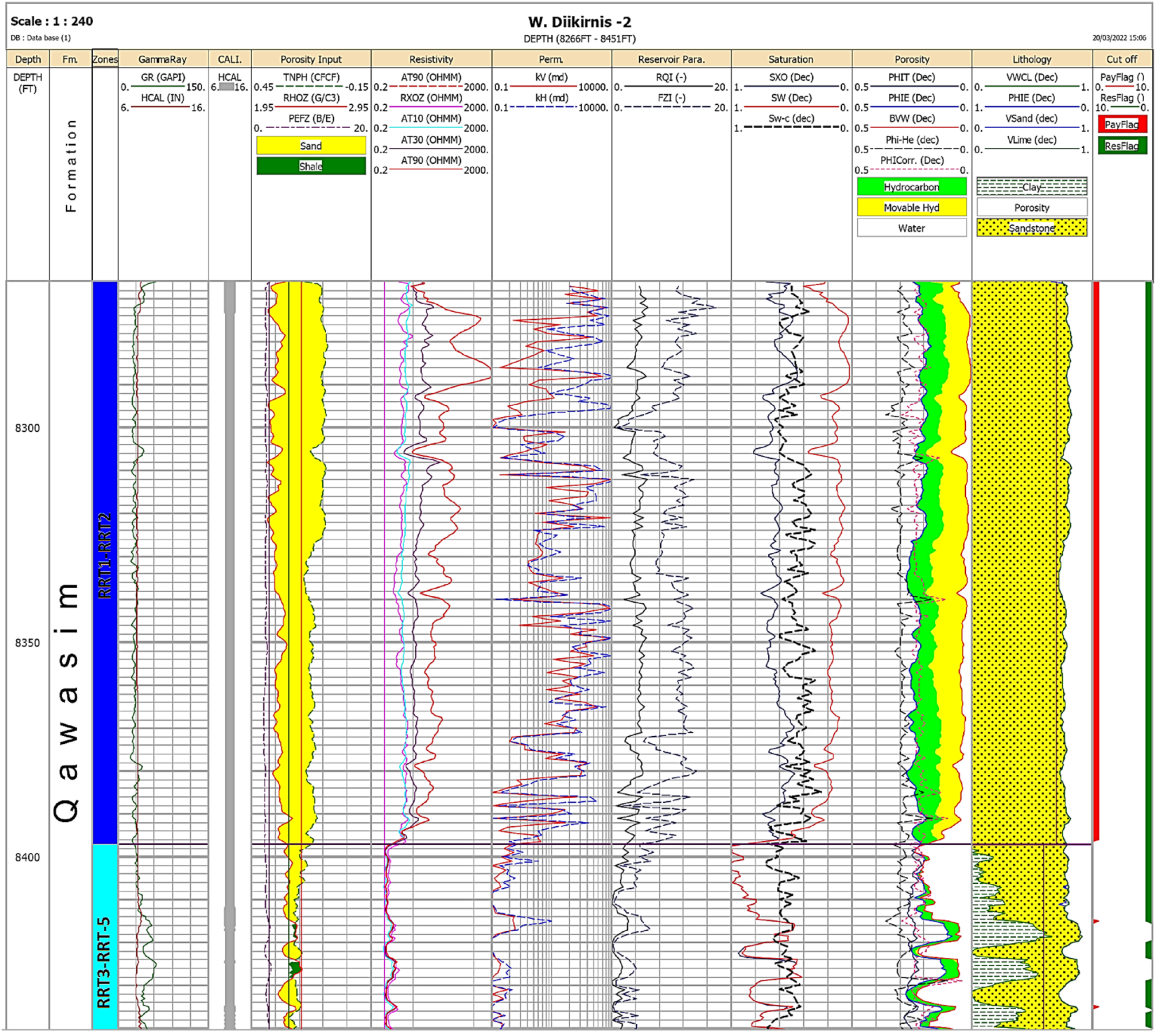
Litho-saturation cross-plot of the WD-2 well, Qawasim reservoir, West Dikirnis Field, Nile Delta, Egypt. Note: GR is the gamma-ray, HCAL is the caliper, TNPHI is the neutron porosity, RHOZ is the bulk density values, PEFZ is the photoelectric factor values, RXOZ is the resistivity of the flushed zone, AT10 is the shallow resistivity, AT30 and AT60 are medium resistivities, AT90 is the deep resistivity, k_V_ and k_H_ are the vertical and horizontal permeabilities, RQI and FZI are the reservoir quality index and the flow zone indicator, respectively, SXO and SW are the fluid and water saturations in the flushed and deep zones, PHIT, PHIE, BVW, PHI-He, PHI-Corr are the total porosity, effective porosity, bulk volume of water, helium porosity and corrected porosity, respectively, VCL, VSand and VLime are the clay, sand and lime volumes, PayFlag and ResFlag are the pay and reservoir zones.

**Figure 7:**
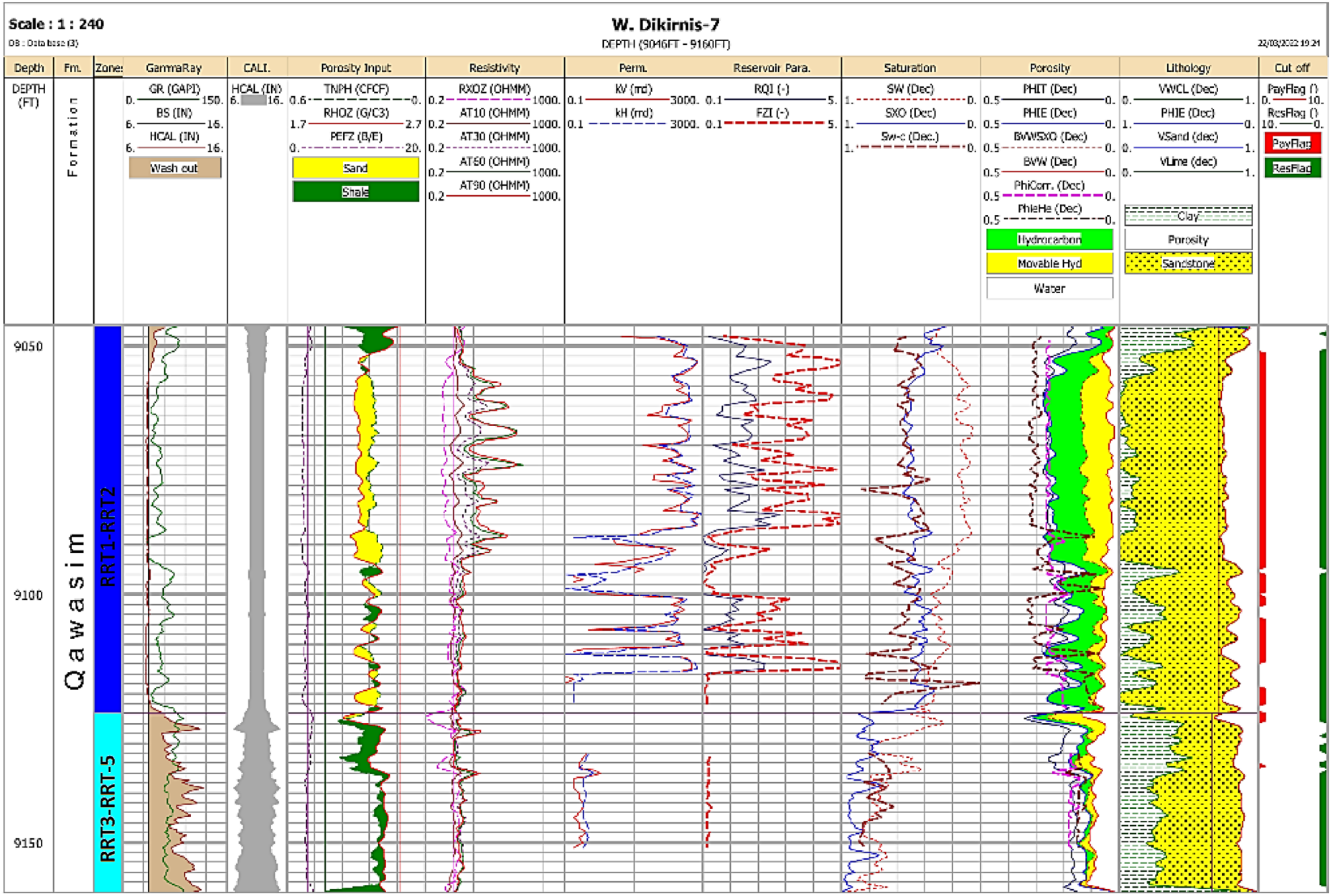
Litho-saturation cross-plot of the WD-7 well, Qawasim reservoir, West Dikirnis Field, Nile Delta, Egypt. Denotations are shown in Fig. 6.

The vertical plot of the petrophysical parameters of the Qawasim oil reservoir states that the studied Qawasim reservoir gross thickness varies from 55 ft in WD-3 well to the southwest of the field up to 185 ft in WD-2 well to the southeastern parts of the field. The petrophysical evaluation of the studied wells signalizes that the Qawasim siliciclastic reservoir can be subdivided into two units (Fig. 5); (1) upper clastic unit with good reservoir quality, and (2) lower unit with less reservoir quality, i.e., the upper unit has better petrophysical characters comparing to the lower unit (Figs. 6 and 7). The average shale content of the upper unit is represented by negligible shale volume ranging from 0.2% (clean) in the WD-2 well to the southeastern parts to about 9.8% in WD-7 well (to the northeast), while it reaches up to 38.1% in the lower clastic unit. Thereby, in contrast to the lower unit, the upper unit consists of thick almost clean sandstones with low gamma-ray, high neutron porosity and high deep resistivity as show in Figs. 6 and 7.

**Figure 8:**
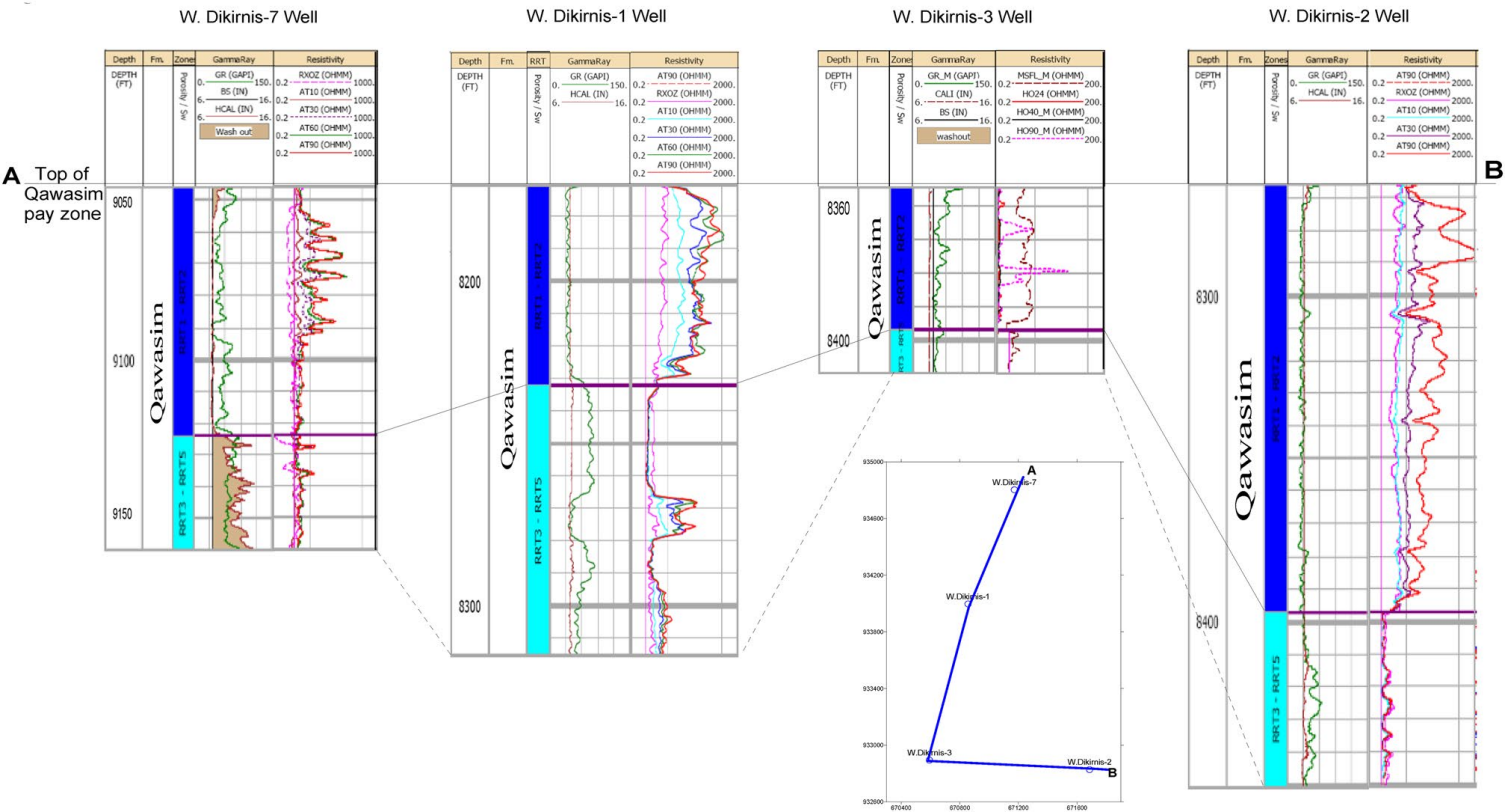
A correlation between for the studied upper and lower units of the Qawasim reservoir, based on the well log data of the different wells at the West Dikirnis Gas Field. Note: GR is the gamma-ray; HCAL is the caliper; RXOZ is the resistivity of the flushed zone; AT10 is the shallow resistivity, AT30 and AT60 are medium resistivities, and AT90 is the deep resistivity.

Besides, the correlative study between the different well in a profile extending from the north to the SW and then the SE direction indicates the highest pay and gross thickness to the SE at WD-2 well, with the highest deep electric resistivity values assigned to the upper unit (Fig. 8). However, thickness is highly reduced in WD-3 well, which may be attributed to a depositional setting with a relatively high stand situation. Also, low gamma-ray sandstone beds with relatively high deep resistivity values were assigned in WD-1 and WD-7 wells with thickness up to 36.5 ft at WD-1 well (Fig. 8, Table 1). It is also indicated that the lowest gross thickness is assigned in WD-3 well due to its relatively high (Sw) water saturation that is indicated by a relatively low deep resistivity values and no pay (dry well). This may be due to it is out of the four-way dip closure of the promising structural high in the West Dikirnis Field.

**Figure 9:**
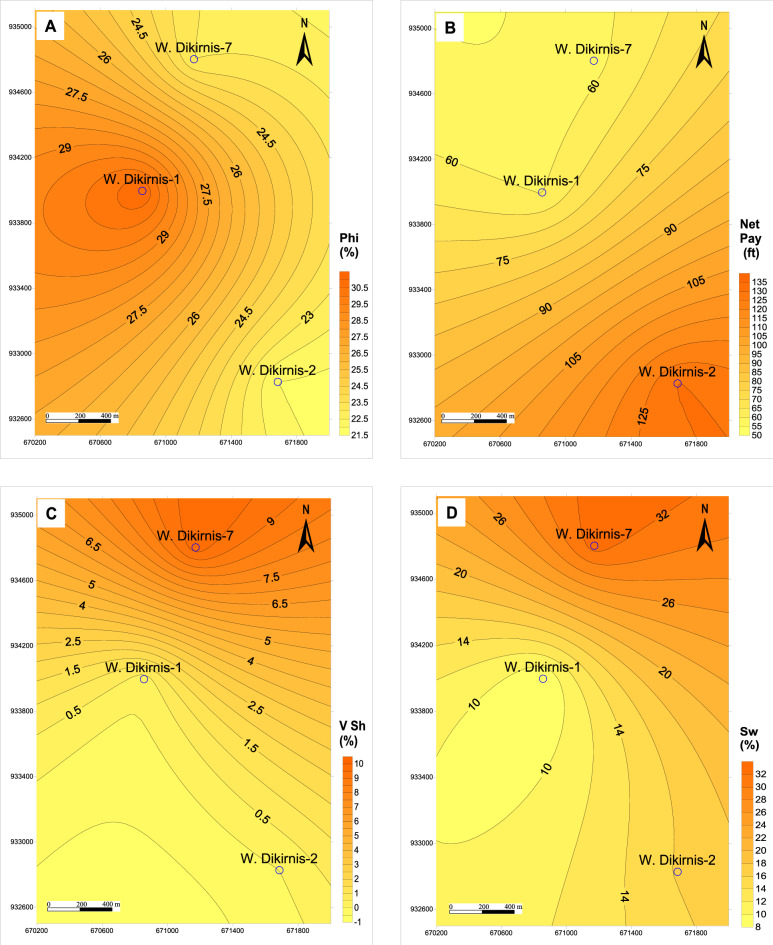
Iso-parametric maps of the upper zone (RRT1-RRT2) of the Qawasim reservoir in West Dikirnis Field, Nile Delta, Egypt, (**A**) Effective porosity, (**B**) net pay thickness, (**C**) Shale volume, and (**D**) Water saturation.

Plotting the petrophysical properties of the studied wells in a spatial distribution represented by a set of iso-parametric maps are based on only three wells which are productive wells, while the fourth well is dry. The spatial distribution of the reservoir parameters of the upper unit indicates that the effective porosity increases to the west and the center of the structural closure of the field (Fig. 9A) according to the tension forces that stretched the reservoir to the borders of the structure/field; however this stretching was not so intensive to cause a wide range of effective porosity which is limited between 22.0 and 31.0% and is considered very good to excellent values. Also, the net-pay thickness increases to the SE of the field (Fig. 9B) with thickness reaches up to 130.3 ft due to decreasing the shale volume (V_Sh_) and the water saturation (Sw) to the SE (Fig. 9C, D). Thereby, the reservoir quality-reducing factors including Sw and V_Sh_ are not effective to the SE, while the gross thickness as an important parameter increases to the SE of the structure to support increasing the prospectivity in this direction.

**Figure 10:**
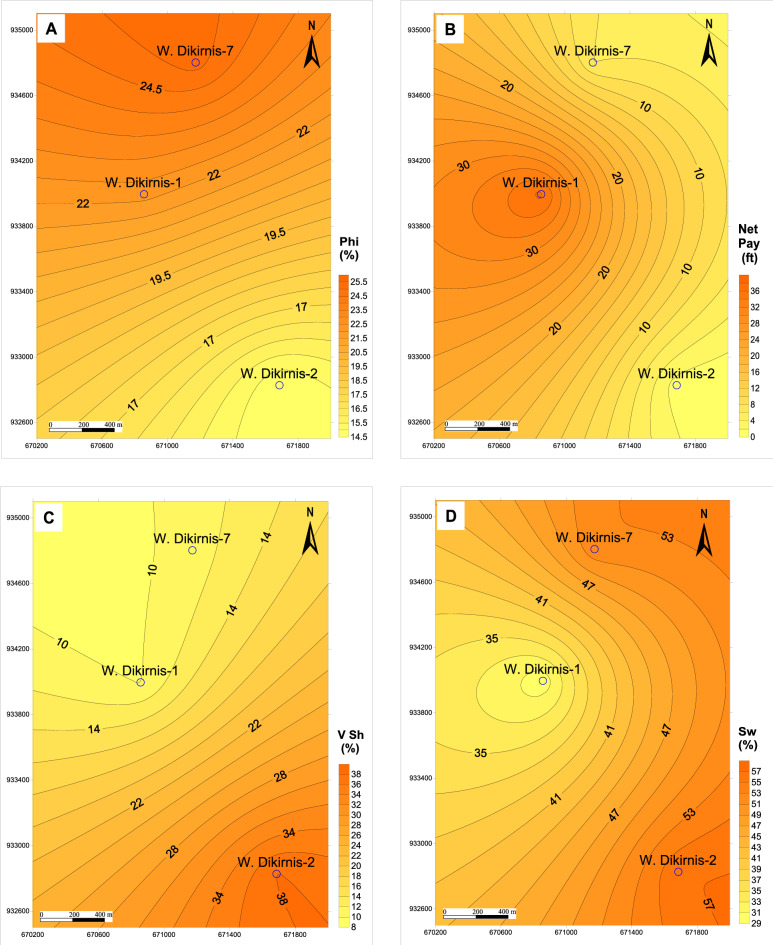
Iso-parametric maps of the lower zone (RRT3-RRT5) of the Qawasim reservoir in West Dikirnis Field, Nile Delta, Egypt; (**A**) Effective porosity, (**B**) net pay thickness, (**C**) Shale volume, and (**D**) Water saturation.

Downward, presenting the spatial distribution of the reservoir parameters for the lower unit of the Qawasim reservoir states that the effective porosity increases to the NW and the center of the field (at WD-1 and WD-7 wells, Fig. 10A), while the net-pay thickness increases to the center of the closure four way structure at (WD-1 well, Fig. 10B). This can be attributed to decreasing the shale volume in this direction and its increase to the SE direction at WD-2 well (Fig. 10C). In this concern, the water saturation, as a reservoir quality-reducing factor, decreases toward the west and center of the field (Fig. 10D) supporting the good reservoir quality to the center of the closure. Increasing the gross and net-pay thicknesses of the lower reservoir unit in the center of the structure and its change upward away from the core of the structure and increasing the reservoir quality upward to the upper unit is due to more compaction effect on the core of the four way closure structure and stretching effect on the top parts and crest of the structure.

Also, increasing the reservoir quality upward is due to changing the depositional environmental setting of the lower unit as prodelta and distal front into the proximal delta-front and delta plain depositional environments of the upper unit with increasing the implementation of the active closure structure as stated by many authors^4^ Shehata et al.^12^).

For more details, the two reservoir zones of the Qawasim Formation were studied petrophysically based on their core data and petrographically based on thin sections. Accordingly, the two zones are composed of four petrographical microfacies, and five petrophysical static rock types (PSRTs). A full description of these microfacies and PSRTs will be discussed as follows.

### Sedimentary facies analysis

#### Lithofacies

Based on the core description, the lithostratigraphy of Qawasim Formation varies significantly in composition between laminated mudstones/siltstones, ripple laminated sandstones, cross-laminated sandstones, cross-bedded sandstones, massive sandstones, conglomerates, and some streaks of fossiliferous shallow marine limestone. These diverse associations of lithofacies are representative to the pre-Messinian salinity crisis sediments which have been deposited in a dominant coarse-grained braided delta. This can be clearly detected from shallowing upward system and transition from the prodelta to the delta-plain settings passing through distal and proximal delta-front deposits (Figs. 2, 5)^4^.

Based on the core description, the clastic sequence of the Qawasim reservoir can be divided into two main depositional units (upper and lower units) (Fig. 5). The upper clastic unit consists of cross-bedded sandstones, massive sandstones and conglomerates primarily deposited in a proximal delta-front and delta-plain, changes into ripple laminated sandstones, cross-laminated sandstones, and laminated mudstones/siltstones lower unit that have been deposited in distal and prodelta setting (Fig. 5).

#### Microfacies

I. **Sandy siltstone/mudstone microfacies**

**Figure 11:**
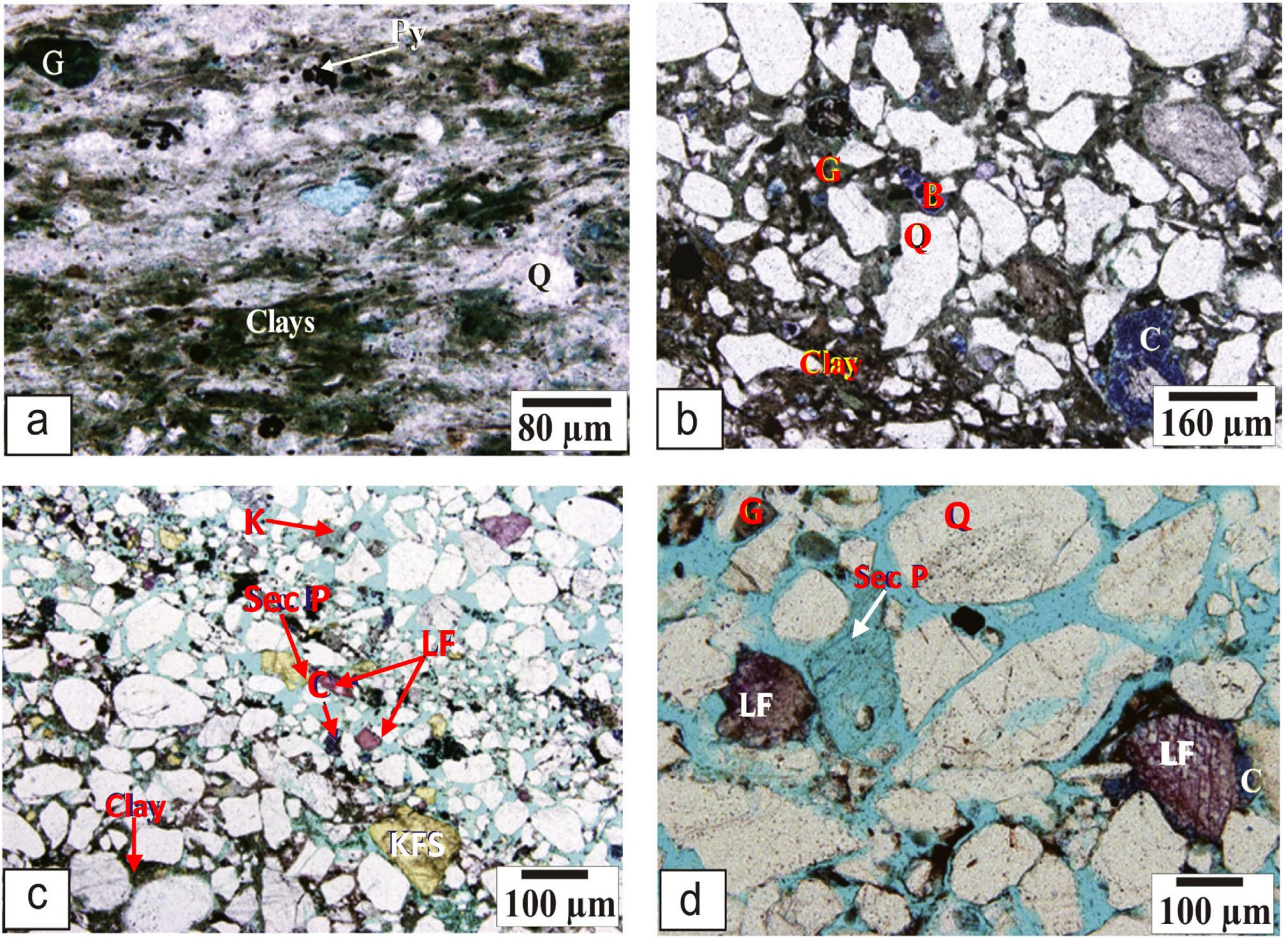
Photomicrographs representing the different petrographical features of the Qawasim Formation showing: (**a**) sandy siltstone/mudstone microfacies (prodelta), (**b**) glauconitic quartz wacke microfacies (distal delta front), (**c**) subarkose arenite microfacies (proximal delta front), and (**d**) sublithic arenite microfacies (delta plain). Mineral abbreviations; Calcite (C), Quartz (Q), K-feldspar (KFS), Lithic fragments (LF), Bioclastics (B), Glauconite (G), Pyrite (Py), Chlorite (Ch), Kaolinite (K), Secondary porosity (Sec. P).

This facies is composed of intercalations of sandy mudstones, siltstones and mudstones. Petrographically, it consists of very fine-grained quartz grains (monocrystalline) (≈ 12.8%) associated with a few glauconite detrital grains that are scattered within clayey and silty matrix dominated as an argillaceous material (Fig. 11a). Pyrite with percentage reaching 4.5% has been also noticed scattered in the framework of this microfacies (Fig. 11a). The presence of pyrite is a good indication for dominance the reducing environments which are suitable for hydrocarbon preservation. A variable amount of the pore spaces (5–25%) are encountered in this microfacies; it can be described as: (1) matrix porosity, and (2) micro vugs. However, porosity of this microfacies is mostly reduced due to the sever compaction and the dominance of the argillaceous matrix. Depositionally, this microfacies is developed in a prodelta sub-environment setting.

II. **Glauconitic Quartz wacke microfacies**

Petrographically, this microfacies consists primarily of angular to sub-angular poorly sorted vari-sized grains ranging in size from very fine to medium-grained quartz displaying poorly sorting texture. The phenoclastic framework of this microfacies encompasses monocrystalline quartz grains and glauconite with a few bioclasts (Fig. 11b). The quartz grains percentage reaching 80.5% and the feldspars are presented by 5% while the lithic fragments are rarely noticed with percentage does not exceed 3%. Well rounded and very fine glauconite pellets are also encountered (11.5%), sometimes present as filling the pore spaces. These clasts are embedded on matrix-supported groundmass that is dominated by argillaceous matter. A considerable amount of pseudo sparry calcite cement has been presented as patches scattered in the wacky groundmass (Fig. 11b). The pore spaces can be distinguished and described as: (1) microfractures, and (2) matrix porosity. Due to the variable implication of the diagenetic process, in particular cementation, compaction and the authigenic pyrite and clay minerals, the porosity values are variable between 10% (poor) to 30% (excellent). Compaction is revealed from the compacted fabric with point, straight and concave-convex contacts. This microfacies is thought to be dominated in a distal delta environment, a commonly in distal sand-bars setting.

III. **Subarkose arenite microfacies**

This microfacies consists of medium to coarse-grained, subrounded to well-rounded, and poorly-sorted grains. The framework of this microfacies is primarily composed of major quartz grains with considerable amounts of feldspars and lithic fragments (Fig. 11c). The quartz grains represent about 85–87% of the total grains, while the feldspar content reaches 7% and the lithic fragments ranging from 6 to 8%. These grain constituents are embedded in equant sparry calcite cement with some pseudo sparite patches. Also, variable amounts of argillaceous matrix are present as scattered clay patches represented primarily by kaolinite forming the groundmass and as filling materials for the pore spaces (Fig. 11c). This microfacies is highly porous (25–30%) and its pore spaces are described as: (1) micro to meso vugs, and (2) micro to meso intergranular pore. It microfacies is deposited in a proximal delta front commonly in the channel fill and mouth-bar sub-environment.

IV. **Sublithic arenite microfacies**

This microfacies is considered the best quality in the Qawasim reservoir sequence. It consists of moderately-sorted coarse- to medium-grained sandstone. The clastic framework of this microfacies is built of quartz grains reaching 78% of the total grains counting, while the lithic fragments reach 16% with a few feldspars content (6%, Fig. 11d). The quartz grains are primarily monocrystalline but sometimes are polycrystalline, while the majority of the lithic fragments are represented by carbonate fragments. These components are embedded in well-developed pseudo sparry calcite attaching the grain components to each other in the form of gravity and meniscus cement (Fig. 11d). Influence of compaction and cementation on this microfacies is very slight and ineffective, so porosity of this microfacies is very high and reaches up to 30%. The pore spaces are distinguished and described as: (1) micro to meso intergranular pore, and (2) meso and micro vugs. This microfacies is predominantly deposited in a delta plain setting, particularly in natural-levee and distributary-channels.

### Petrophysical conventional core data

For more details, the borehole scale petrophysical well logging studies have been supported by plug scale conventional core studies which indicate the need to distinguish the rock samples into five reservoir rock types: RRT1 (138 samples), RRT2 (95 samples), RRT3 (114 samples), RRT4 (30 samples), and RRT5 (58 samples). Among these, the first and second RRTs represent the upper unit of the Qawasim Formation and both are characterized by bulk density ranging from 1.670 to 2.240 g/cm^3^, helium porosity ranging between 15.8 and 36.2%, and permeability ranging from 150 md up to 10,011 md (Table 2).

**Table 2:** The petrophysical and reservoir quality parameters and their ranks derived from the core data of the Qawasim Formation in wells (WD-2, WD-3, and WD-7), West Dikirnis Oil Field, Nile Delta of Egypt.

RRTs	Depositional environment	Microfacies		DRT	ρ_b_	ρ_g_	∅_He_	∅_FS_	k_V_	k_H_	λ_k_	Sw	So	Sg	RQI	FZI	RPI	Reservoir
					(g/cc)	(g/cc)	%	%	md	md		%	%	%	µm	µm	rank	ranks
RRT1 (N = 138)	Prodelta and distal delta front	Sublithic arenite	Min	15.0	1.760	2.609	17.1	15.8	767.6	1600	0.87	27.90	0.00	28.99	3.04	8.88	3.0	Good
			Max	16.0	2.196	2.680	33.6	36.3	9949	10,011	1.57	62.20	20.0	70.10	5.66	15.00	2.0	Very good
			Mean	15.5	1.882	2.639	28.6	25.5	5609	6592	1.10	43.85	7.20	48.95	4.71	11.75	2.0	Very good
RRT2 (N = 95)	Prodelta and distal delta front	Subarkose arenite—Sublithic arenite	Min	13.9	1.670	2.610	15.8	9.5	150	440	0.86	25.17	0.00	28.70	1.66	5.13	3.0	Good
			Max	15.5	2.240	2.710	36.2	36.0	5565	5732	2.33	70.40	15.30	68.00	4.24	11.30	2.0	Very good
			Mean	14.6	1.880	2.647	29.0	24.9	1854	2686	1.28	46.06	5.48	48.47	2.92	7.38	3.0	Good
RRT3 (N = 114)	Proximal delta-front & delta plain	Subarkose arenite—Glauconitic quartz wacke	Min	12.1	1.779	2.600	15.4	12.3	32.4	80.6	0.82	28.40	0.00	2.90	0.65	2.14	5.0	Poor
			Max	15.1	2.267	2.710	32.5	31.5	1356	1666	2.28	85.40	19.0	69.10	2.50	9.56	3.0	Good
			Mean	13.5	1.903	2.652	28.2	23.6	443.7	708.1	1.34	49.64	5.01	45.35	1.57	4.45	4.0	Fair
RRT4 (N = 30)	Proximal delta-front & delta plain	Glauconitic quartz wacke	Min	11.4	1.830	2.642	22.6	11.3	5.70	43.90	1.69	30.40	0.00	28.76	0.44	1.50	5.0	Poor
			Max	13.5	2.098	2.710	30.8	32.7	221	1133	4.75	71.24	9.70	68.40	1.90	4.27	3.0	Good
			Mean	12.6	1.944	2.667	27.1	21.2	46.9	375.2	3.18	46.69	1.67	51.65	1.09	2.87	4.0	Fair
RRT5 (N = 58)	Proximal delta-front & delta plain	Sandy mudstone/Siltstone	Min	7.2	1.935	2.638	6.5	10.3	0.030	0.085	0.88	29.81	0.00	13.30	0.04	0.18	6.0	Tight
			Max	12.0	2.534	2.720	27.5	31.0	38.9	40.8	4.85	86.70	7.20	69.20	0.38	2.01	5.0	Poor
			Mean	9.1	2.190	2.666	17.8	19.0	3.31	7.75	1.57	61.81	0.74	38.35	0.14	0.56	6.0	Tight

where RRTs are the reservoir rock types; N is the number of available samples; DRT is the discret rock type; ρ_b_ & ρ_g_ are the bulk and grain densities, respectively; ∅_He_ & ∅_FS_ are the helium and fluid summation porosity, respectively; k_V_ and k_H_ are the vertical and horizontal gas permeability; λ_k_ is the permeability naisotorpy; SW, So and Sg are the water, oil and gas saturations, RQI and FZI are the reservoir qulaity index and the flow zone indicator, respectively; and RPI rank, is the reservoir ponteility index. The RPI ranks are based on the classification ranks of the reservoir quality parameters (Nabawy^32^^,^^48^; Nabawy et al.^41^; Elgendy et al.^50^).

Though the presence of some water-bearing zones (Sw ≥ 65%), these two rock types are characterized by average water saturation less than 50%, gas saturation of averages 48–49%, while the oil/condensates saturations average 5.48–7.20% (Table 2).

Therefore, the reservoir quality parameters of these high quality rock types indicate good to very good quality of averages (average RQI = 4.71 μm, average FZI = 11.75 μm, and average RPI rank = 2.0 for the RRT1 samples, while for the RRT2 samples the average RQI = 2.92 μm, av., FZI = 7.38 μm, and average RPI rank = 3.0, Table 2).

Furthermore, the RRT3-RRT-5 samples are dominated in the lower prodelta and distal delta unit which has less reservoir quality (tight to good quality) than the upper unit. These rock types are characterized by poor to excellent porosity (6.5 ≤ ∅ ≤ 32.5%), whereas the permeability varies from tight (av. k = 3.31 md) to very good permeability values (av. k = 708.1 md, Table 2). The average water saturation of these rock types varies from 46.7 to 61.8%, whereas the average gas saturations vary between 37.46 and 51.65% for these three rock types. So, the RQI varies from 0.04 μm for the RRT5 to 2.50 μm for the RRT3, the FZI varies between 0.18 and 9.56 μm, and the RPI ranks vary between tight and good reservoir quality. The petrophysical characters and reservoir quality of the RRT3 samples are considered transitional between the RR1-RRT2 of the upper unit and the RRT4-RRT5 of the lower unit.

Due to the variation of the reservoir quality between the Qawasim rock types, their average discrete rock type values (DRT) varies from 15.5 for the RRT1 to 9.1 for the RRT5. The permeability anisotropy varies intensively between 0.82 (for RRT3 samples indicating presence of some micro fractures supporting the vertical flow) and 4.75 (for the RRT4 samples indicating presence of some shale streaks opposing the vertical flow).

The variation in anisotropy and reservoir quality of the Qawasim sequence is due to the noticeable variations in the mineral composition and different depositional settings between the proximal delta-front and the distal delta/prodelta.

## Discussion

### Reservoir zonation

**Figure 12:**
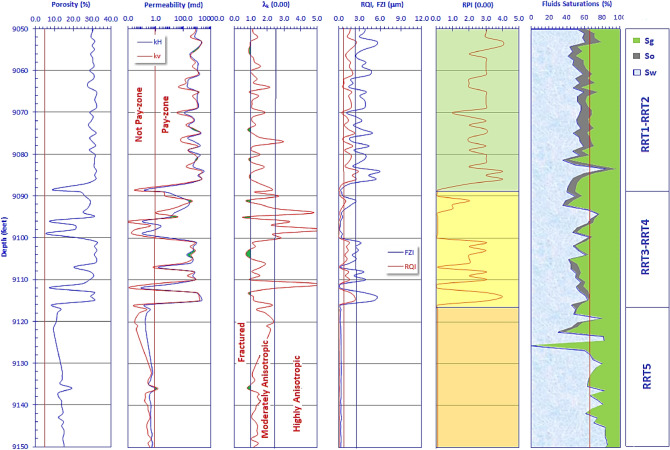
Vertical matching of the core data of WD-7 well including the helium porosity, permeability (k_V_ and k_H_, vertical and horizontal; respectively), permeability anisotropy (λ_k_), reservoir quality indices (RQI), flow zone indicators (FZI), reservoir potentiality index (RPI), as well as water, oil and gas saturations (Sw, So, Sg, respectively) versus depth.

The Qawasim clastic sequence is typically a coarsening upward sequence of fluvial deltaic characters. Due to the changing upward grain size, the sequence composition changes upward from laminated mudstones/siltstones at the base to the coarse and very coarse sandstone at the top of the reservoir sequence. Consequently, the petrophysical properties of the studied samples increases upward from relatively poor to fair reservoir quality at the base of the sequence to much better quality at the top of the sequence. However, the reservoir quality of Qawasim Formation at its base in WD-7 well is relatively tight to poor due to the dominance of low porosity and permeability values (Fig. 12). The vertical plot of the conventional core data of WD-7 well indicates that porosity fluctuates around 30% from the top of the cored interval down to depth 9089 ft. This fluctuation becomes much spikier down to depth 9117 ft fluctuating between 8 and 30%, and then moves down touching the porosity cutoff value (5%, Fig. 12). In this concern, dividing the studied samples into some RRTs was critically needed to get a precise mathematical model to estimate and predict the petrophysical and parameters of reservoir quality of other similar sequences in the neighborhood. The upper sequence unit is composed entirely from the sublithic arenite (RRT1) samples and the subarkose arenite (RRT2) samples, while the middle units is composed primarily from RRT3 and RRT4 besides to some samples from the other RRTs; it is relatively highly heterogeneous. This spiky petrophysical behavior is attributed to the occurrence of some microfractures (λ_k_ < 1.0, track 3, shaded green, Fig. 12), and shale streaks (λ_k_ > 2.5). It is well-known that, increasing the horizontal permeability than the vertical one of a given sample (so λ_k_ > 1.0) is due to its primary depositional nature and presence of some shale streaks which increases the value of λ_k_ to more than 2.5. On the other side, increasing the vertical permeability than the horizontal one is due to presence of some vertical microfractures due to the dominance of overload stresses; thereby λ_k_ is less than 1.0^33,41,48–50^.

Eventually, the lower unit is mostly not promising and is composed of the sandy mudstone/siltstone RRT5 samples. The muddy and silty composition of the base of Qawasim Formation in WD-7 well reduced both the vertical and horizontal permeabilities to be less than 1.0 mD. However, the base of Qawasim Formation in the other wells are considered of poor to fair quality base on the classification ranks of Nabawy et al.^37,38,40,41^.

On the other side, permeability values of the upper unit are considered very high reaching to thousands mD due to their pebbly and the coarse sand composition.

The variation of the reservoir properties between the different levels caused a somewhat spiky behavior of the values of RQI and FZI and the RPI ranks, especially through the middle unit. The reservoir quality variation between these three units can be also attributed to the variation in the pore radius, so some oil residues are primarily detected in the upper unit, with a few content in the middle unit, while no oil residues is detected in the micro and nano pores of the lower unit (Last track, Fig. 12). The presence of a relatively high water saturation along the entire sequence of the reservoir (25.0–85.4%, Table 2) is due to the relatively high shale content.

### Implementation of the bulk density by the grain density and porosity

**Figure 13:**
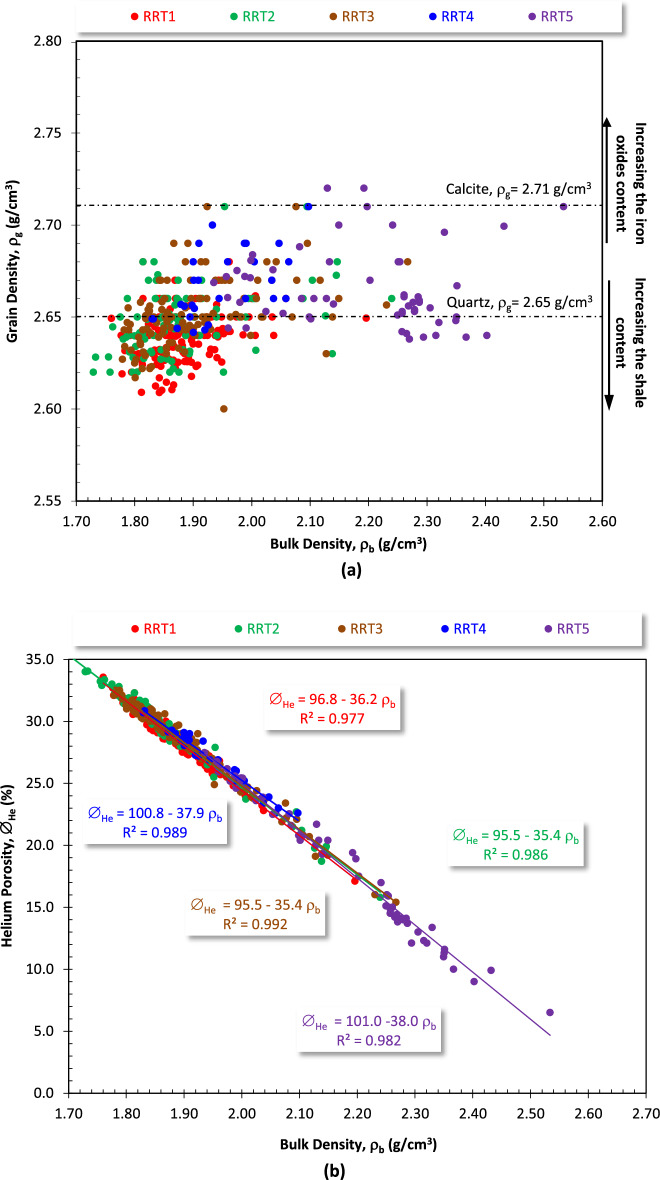
Plotting the bulk density (ρ_b_) versus (**a**) the grain density (ρ_g_), and (**b**) the helium porosity (∅_He_) of the Qawasim reservoir.

The grain density and pore spaces are the major contributors to the bulk density, so plotting the bulk and grain densities versus each other of the different rock types indicates a wide range of both the bulk and grain density values (1.670–2.534 g/cm^3^, and 2.60–2.72 g/cm^3^, respectively, Fig. 13a, Table 2). It seems that most of the sublithic arenite RRT1 samples are characterized by grain density less than 2.65 g/cm^3^ due to the presence of slight clay content and some isolated pore spaces. Increasing the grain density values of some samples are due to increasing the iron oxides and heavy mineral content, primarily pyrite as indicated from the petrography. The wide range of the bulk density values are due to the tightness of the dense sandy mudstone/siltstone RRT5 samples, and the low density sublithic and subarkose arenite RRT1 and RRT2 samples.

The bulk density-porosity plot has a wide application as a quality control for the calculated petrophysical core dataset^28,29,51,52^. For the present study, a set of highly reliable models were obtained for this relationship (R^2^ ≥ 0.977, Fig. 13b) which indicates a high quality petrophysical measurements. With the except of the RRT1 arenite samples, the multiplication factors and the constants of this set of models decreases with increasing the reservoir quality from the laminated siltstone/mudstone RRT5 sandy samples to the RRT2 coarse sandstone samples. Due to the high reliability of this set of models, a general model can be established for calculating the porosity from the bulk density of these set of fluvial facies.

11 $$ \emptyset_{{{\text{He}}}} = {96}.{6} - {36}.0\uprho _{{\text{b}}} \;\left( {{\text{R}}^{{2}} = 0.{99}0} \right) $$

### Impacts of the fluid barriers on the anisotropy of permeability

The permeability represents the ability of the reservoir to transmit fluids in both vertical and horizontal directions. To avoid the water breakthrough during production, it is desired to get much higher permeability in the horizontal direction than that in the vertical direction, i.e., to get high permeability anisotropy. This high anisotropic permeability (λ_k_ > 1.0) refers to presence of some barrier streaks, while the relatively low permeability anisotropy (λ_k_ < 1.0) refers to the presence of some microfractures.

**Figure 14:**
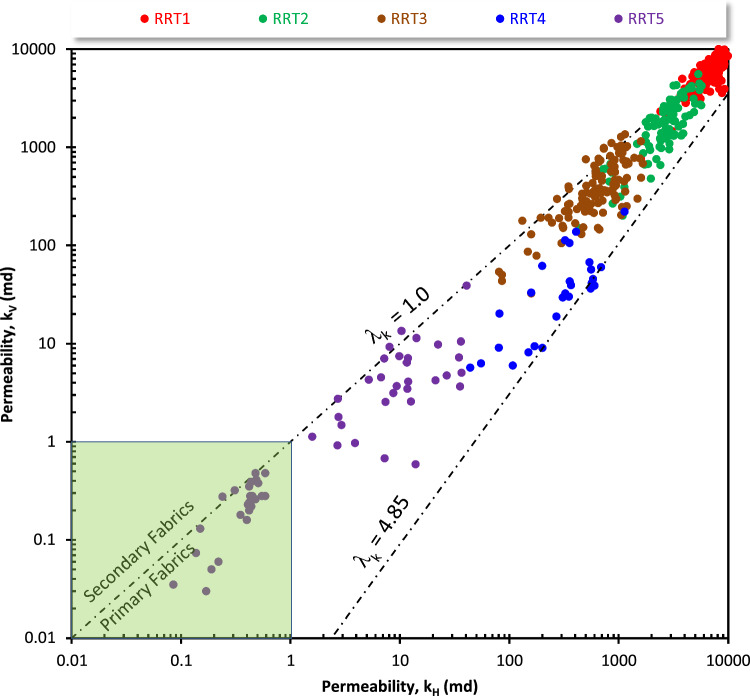
Plotting the vertical permeability as a function of the horizontal one as a tool for discriminating the samples into secondary and primary-bearing samples and the range of the permeability anisotropy (λ_k_). Shaded green area represents the non-prospective samples (k < 1.0 mD).

For the Qawasim reservoir, plotting the horizontal permeability versus the vertical permeability that only few samples of the sandy mudstone/siltstone RRT5 samples are not promising (k < 1.0 mD, tight samples, Fig. 14). It is indicated that the permeability anisotropy (λ_k_) increases from the sublithic arenite RRT1 samples to the RRT5 samples, where the anisotropy reaches up to 4.85 for the glauconitic quartz arenite RRT4 samples. Microfractures are only assigned to a few samples from the different RRTs samples, where slight to moderate secondary anisotropy (1.0 > λ_k_ > 0.82, Fig. 14) values are assigned to the RRTs samples except for the RRT4 (av. λ_k_ = 3.18). The relatively high λ_k_ of the RRT4 samples could be attributed to the presence of many barrier streaks, as referred by the anisotropy classification of Nabawy^33^, due to their proximal delta-front depositional setting and dominance of shale streaks.

Thereby, the dominance of the depositional primary fabrics in Qawasim Formation and its deltaic setting indicates that production will be carried out in a radial steady flow regime with no disturbance.

### Implementation of porosity on k_H_ and k_V_

**Figure 15:**
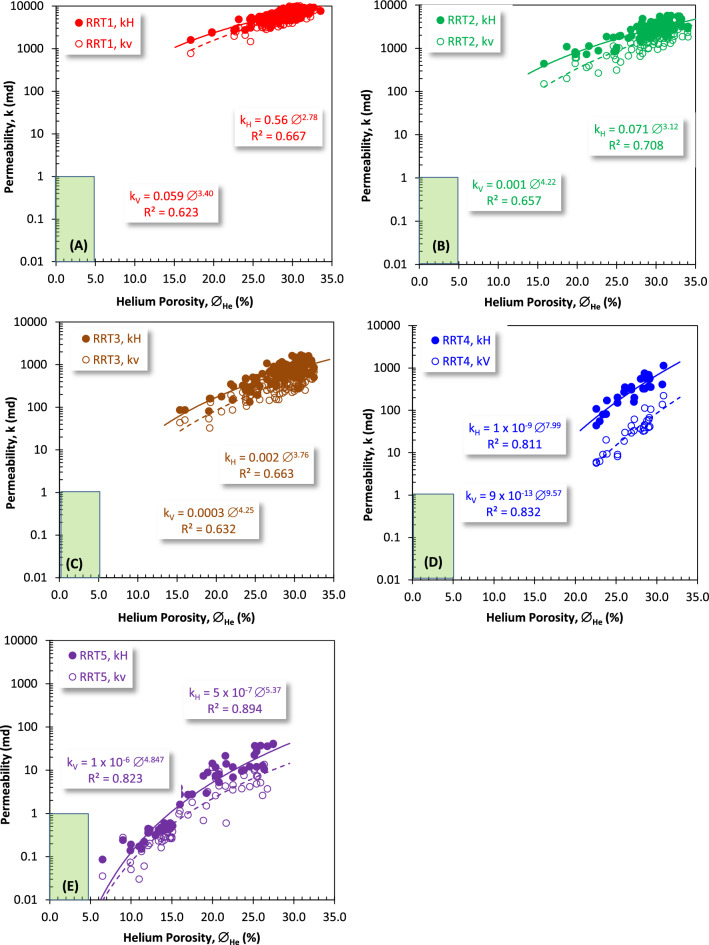
Plotting the vertical and horizontal permeabilities (k_V_ and k_H_, respectively) versus the helium porosity (∅_He_) for the different rock types: (**A**) RRT1, (**B**) RRT2, (**C**) RRT3, (**D**) RRT4, and (**E**) RRT5 samples of the Qawasim reservoir, West Dikirnis Oil Field, Nile Delta, Egypt.

Rock typing is mostly based on the ability of porosity to contribute to the permeability. However due to the difference in the spatial distribution of pore spaces and authigenic clay minerals, this contribution varies between the vertical and the horizontal directions. In this concern, plotting permeability of the Qawasim reservoir in both directions as a function of porosity (Fig. 15) indicates that the sublithic arenite (RRT1) and the subarkose arenite (RRT2) have the best rock types with most permeability values higher than 1000 mD (Fig. 15A,B). This rock typing has been applied with the support of the average values of discrete rock type, as recommended by many authors^39,45,53–55^, which equal to 15.5 and 14.6 for the RRT1 and RRT2 sublithic and subarkose arenite samples, respectively. This plot also indicates that the highest difference and separation between the horizontal and vertical permeabilities are assigned to the RRT3 and the RRT4 samples (Fig. 15C,D). The highest reliability of this relationship was assigned to the laminated sandy mudstone/siltstone and the glauconitic quartz wacke RRT4 and RRT5 samples (0.894 ≥ R^2^ ≥ 0.811, Fig. 15D,E). These highly reliable relationships are due to increasing shale content and the laminated mudstones/siltstones nature of the RRT4 and RRT5 samples.

### The main reservoir quality attributes

**Figure 16:**
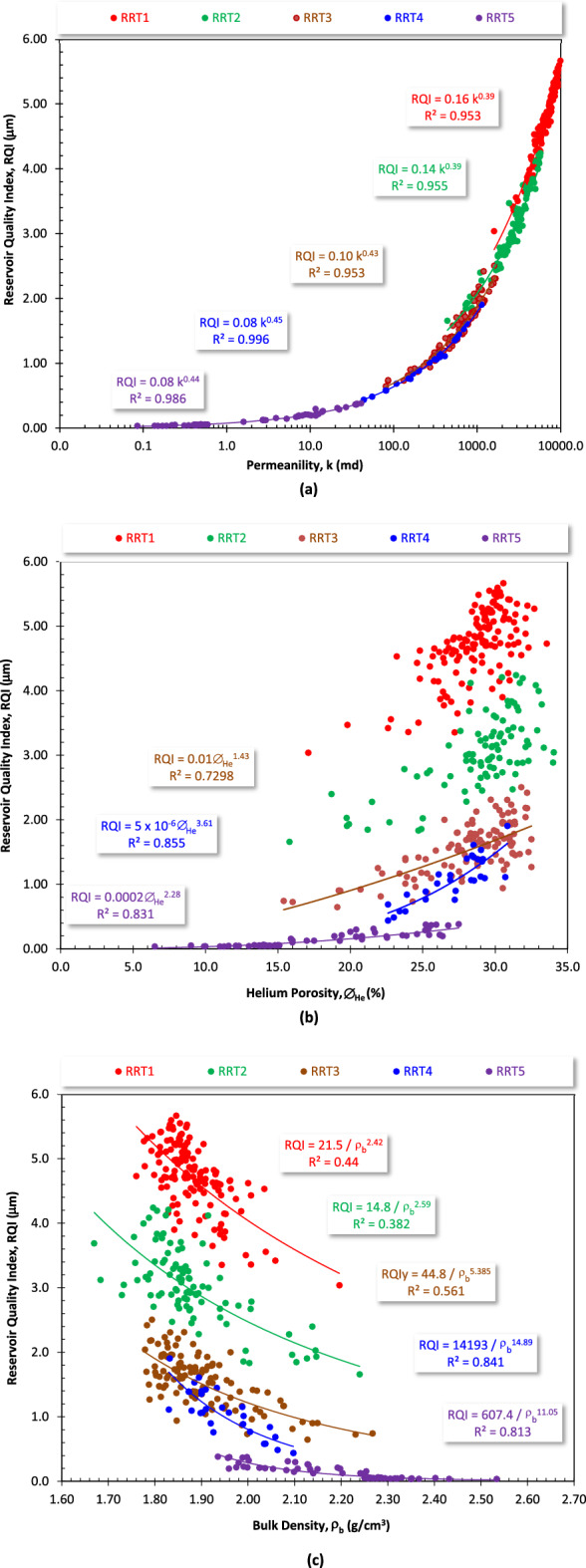
Plotting the reservoir quality index (RQI) as a function of (**a**) the permeability (k), (**b**) the helium porosity (∅_He_), and (**c**) the bulk density (ρ_b_) for the different reservoir rock types of the Qawasim reservoir, West Dikirnis Oil Field, Nile Delta, Egypt.

According to the reservoir quality classification ranks of Nabawy et al.^37,38^ (Table 3), the values of reservoir quality index of the Qawasim RRT1 and RRT2 arenite samples are considered very good to excellent quality (2.0 μm ≤ RQI), poor to good for the RRT3 and RRT4 samples (0.25 ≤ RQI ≤ 2.0 μm), and tight quality for the laminated sandy mudstone/siltstone RRT5 samples (RQI ≤ 0.25 μm). The RQI values, in general, which are primarily attributed by the permeability and by the porosity and the bulk density to some extent (Fig. 16). From this plot, it is indicated that permeability is the major attribute to the RQI values of the different RRTs (R^2^ ≥ 0.953, Fig. 16a). A set of mathematical models was obtained for estimating the RQI in terms of the permeability with the similar multiplication factors (0.08–0.10) and the exponents (0.43–0.45) for the proximal delta-front and delta plain RRT3, RRT4 and RRT5 laminated sandstone (subarkose arenite and glauconitic quartz wacke microfacies) and the laminated mudstone/siltstone samples. The same similarity was assigned for the prodelta and distal delta front RRT1 and RRT2 massive and laminated sandstone and pebbly sandstone samples (sublithic and subarkose arenite microfacies) (Fig. 16a). Due to the similarity of the exponent values and the multiplication factors, a generalized model was constructed to estimate the RQI in terms of the permeability with very high reliability as follows.

12 $$ {\text{RQI }} = \, 0.0{\text{8 k}}^{{0.{47}}} \;({\text{R}}^{{2}} \ge 0.{997}) $$

**Table 3:** Classification ranks of the petrophysical and reservoir quality parameters including porosity (∅), permeability (k), reservoir quality index (RQI), flow zone indicator (FZI), and reservoir potentiality index (RPI)^33,38,39^.

Porosity %	Permeability md	RQI μm	FZI μm	RPI rank	Reservoir rank
∅ ≤ 5	k ≤ 1	RQI ≤ 0.25	FZI ≤ 1.00	Tight	6.0
5 < ∅ ≤ 10	1 < k ≤ 10	0.25 < RQI ≤ 0.50	1.00 < FZI ≤ 2.50	Poor	5.0
10 < ∅ ≤ 15	10 < K ≤ 100	0.50 < RQI ≤ 1.00	2.50 < FZI ≤ 5.00	Fair	4.0
15 < ∅ ≤ 20	100 < k ≤ 1000	1.00 < RQI ≤ 2.00	5.00 < FZI ≤ 10.0	Good	3.0
20 < ∅ ≤ 25	1000 < k ≤ 10,000	2.00 < RQI ≤ 5.00	10.0 < FZI ≤ 15.0	Very good	2.0
25 < ∅	10,000 < k	5.00 < RQI	15.0 < FZI	Excellent	1.0

In addition to the permeability as a representative parameter for the fluid flow capacity, porosity as a representative parameter for the ability of the reservoir to store fluids is considered another contributor to the quality of reservoir. Dependence of the RQI on the porosity values of the Qawasim Formation is much disturbed than that of the permeability. The reliability of the obtained porosity-RQI mathematical models increases in a trend form the sublithic arenite RRT1 samples (R^2^ = 0.426, Fig. 16b) to the laminated sandy mudstone/siltstone RRT5 (R^2^ = 0.931, Fig. 16b). This disturbance of the porosity attribute is due to variation of the shale content from the massive sandstone RRT1 samples (sublithic arenites) to the laminated sandy mudstone/siltstone RRT5 samples. It can be also attributed to the various mineral composition of the lithic fragments of the sublithic arenites, where the petrophysical properties and the reservoir quality are highly dependent on the

The bulk density as an alternative to the porosity is considered another attribute to the reservoir quality of the Qawasim reservoir, where the RQI decreases with increasing the bulk density in an inverse proportional relationship (Fig. 16c). This can be explained by the fact that increasing the bulk density means decreasing the pore volume and increasing the grain volume, i.e., the pore volume contribution to the RQI decreases.

**Figure 17:**
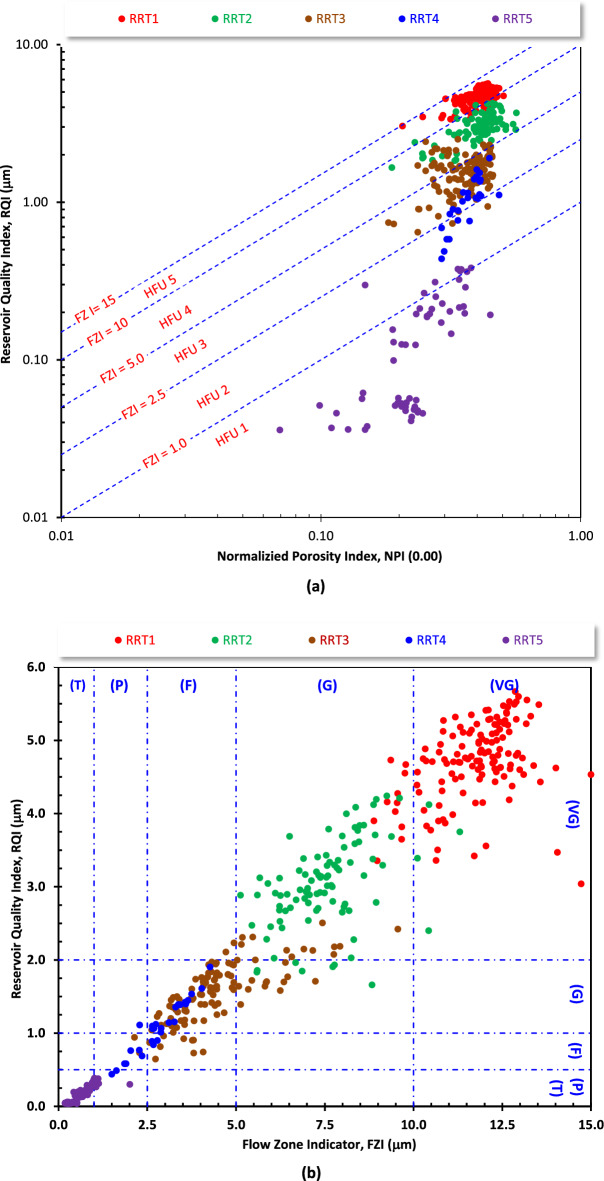
Plotting the reservoir quality index (RQI) as a function of (**a**) the normalized porosity index (NPI), and (**b**) the flow zone indicator (FZI) of the Qawasim reservoir, West Dikirnis Oil Field, Nile Delta, Egypt. VG, G, F, P and T refer to very good, good, fair, poor and tight reservoir qualities. These classification ranks are listed following the classification of Nabawy et al. [37,38].

Plotting the RQI versus the normalized porosity index (NPI) superimposed by the FZI values indicates that, the samples studied can be grouped into five hydraulic flow units (HFUs) (Fig. 17a). The HFU1 is a tight unit (FZI < 1.0 μm) composed primarily from the laminated siltstone/mudstone samples of the RRT5. The HFU2 and HFU3 are characterized by poor to fair reservoir quality (1.0 ≤ FZI ≤ 5.0 μm); they are composed of the laminated sandstone RRT3 and RRT4 samples. The massive sandstone samples (sublithic and subarkose arenite microfacies) of the RRT1 and RRT2 types are primarily clustered in the HFU-4 and HFU-5 which are characterized by good to very reservoir quality (5.0 ≤ FZI ≤ 15.0 μm) following the classification ranks of Nabawy et al.^37,38^.

This plot is supported by plotting the RQI versus the FZI (Fig. 17b) which indicates that the sublithic arenite RRT1 and the subarkose arenite RRT2 samples are representative for good to very good reservoir qualities, while the RRT5 are considered tight and impervious samples. The RRT3 and the RRT4 are considered transitional between the good to very good quality and the tight samples (Fig. 17b).

### Implication of the pore volume on the bulk volume of water

**Figure 18:**
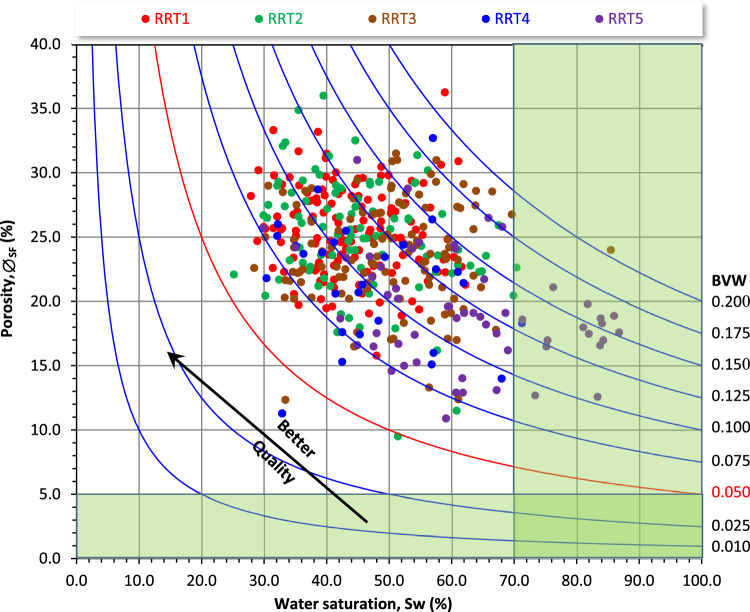
Buckles plot of the bulk volume of water by presenting the summation fluids porosity versus the water saturation. Shaded green area represents the non-prospective samples (∅_cutoff_ = 5.0%, Sw_cutoff_ = 70%). BVW is the bulk volume of water.

Increasing the pore volume is primarily associated with a reduction in the complexity in the pore throat distribution and increasing the pore connectivity which in turns decreases the retained irreducible water saturation, i.e., increases the reservoir quality. Thereby, studying the impact of the pore volume on the water saturation is an important issue in the workflow study for the reservoir quality. This could be achieved by plotting the water saturation as a function of porosity (∅) of the studied Qawasim reservoir (Fig. 18). From this plot, the irreducible water saturation (Sw_irr_) and the bulk volume of water (BVW) can be estimated. The BVW is the multiplication output of the water saturation and the porosity values^56,57^, therefore the ∅-Sw plot is adopted by a hyperbola set of constant BVW. The lower BVW values indicate coarser grain size, higher pore connectivity, and higher permeability values, i.e., much better reservoir quality^38^. Scattering the studied Qawasim RRTs samples are attributed to the variation in their pore fabric complexity and mineral composition. It is also indicated that most samples are characterized by BVW higher than 0.75, with very few samples having BVW less than 0.05, i.e., the hydrocarbons production from the Qawasim reservoir is primarily associated with much water cut. Superimposing this plot with Sw and porosity cutoffs, indicates that most of the Qawasim samples are promising except for some of the laminated siltstone/mudstone RRT5 samples (Fig. 18). Eventually, based on this plot, the irreducible water saturation of the Qawasim RRTs samples is estimated as 30%.

## Conclusions

- The implementation of the microfacies and the reservoir rock types on the reservoir properties of the fluvial sequence of Qawasim reservoir in the Nile Delta were delineated on the borehole and plug scales.
- Petrophysically, based on the borehole data, the Qawasim reservoir was subdivided into two units; the upper unit has a much better reservoir quality than the lower one and has been formed in proximal delta-front and delta-plain setting while the lower unit has been deposited in prodelta and distal front setting. The upper unit is characterized by a very good porosity (22.0–31.0%), considerable net-pay thickness (58.5–130.3 ft), low shale content (0.2–9.8%), and low water saturation (8.1–32.9%).
- On the core plug scale, the proximal delta-front and delta-plain upper unit of the Qawasim reservoir was divided into two reservoir rock types (RRT1-RRT2) while the lower unit of the prodelta and distal front is composed of three RRTs (RRT3-RRT5).
- The RRT1 and RRT2 samples of the upper unit have the best reservoir quality (av. DRT = 15.5, av. ∅ = 28.6%, av. k_H_ = 6592 mD, av. Sw = 43.85%, av. RQI = 4.71 μm, and FZI = 11.75 μm, i.e., the reservoir quality is considered very good), while the RRT5 has the lowest reservoir quality (av. DRT = 9.1, av. ∅ = 17.8%, av. k_H_ = 7.75 mD, av. Sw = 61.81%, av. RQI = 0.14 μm, and FZI = 0.56 μm, i.e., the reservoir quality is considered tight).
- The reservoir quality of these RRTs is due to their depositional deltaic setting and microfacies type, where the RRT1 is primarily composed of sublithic arenite microfacies, while the RRT5 consists of sandy mudstone/siltstone microfacies.
- The RRT2-RRT4 groups are considered transitional between these two rock types (RRT1 and RRT5) and they are composed of sublithic arenites, subarkose arenite, glauconitic quartz wacke, and sandy mudstone/siltstone microfacies.

## Data Availability

The data will be available on demand, contact corresponding author: Bassem S Nabawy; bsnabawy@yahoo.co.uk; bs.nabawy@nrc.sci.eg.
